# Distinctive roles of syntaxin binding protein 4 and its action target, TP63, in lung squamous cell carcinoma: a theranostic study for the precision medicine

**DOI:** 10.1186/s12885-020-07448-2

**Published:** 2020-09-29

**Authors:** Erkhem-Ochir Bilguun, Kyoichi Kaira, Reika Kawabata-Iwakawa, Susumu Rokudai, Kimihiro Shimizu, Takehiko Yokobori, Tetsunari Oyama, Ken Shirabe, Masahiko Nishiyama

**Affiliations:** 1 Department of General Surgical Science, Gunma University Graduate School of Medicine, 3-9-22 Showa-machi, Maebashi, Gunma 371-8511 Japan; 2 Department of Molecular Pharmacology and Oncology, Gunma University Graduate School of Medicine, 3-9-22 Showa-machi, Maebashi, Gunma 371-8511 Japan; 3 Department of Respiratory Medicine, Comprehensive Cancer Center, International Medical Center, Saitama Medical University, 1397-1 Yamane, Hidaka-City, Saitama, 350-1298 Japan; 4 Division of Integrated Oncology Research, Gunma University Initiative for Advanced Research, 3-9-22 Showa-machi, Maebashi, Gunma 371-8511 Japan; 5 Department of Surgery, Division of General Thoracic Surgery, Shinshu University Graduate School of Medicine, 3-1-1 Asahi, Matsumoto, Nagano, 390-8621 Japan; 6 Department of Diagnostic Pathology, Gunma University Graduate School of Medicine, 3-9-22 Showa-machi, Maebashi, Gunma 371-8511 Japan; 7 Gunma University, 3-9-22 Showa-machi, Maebashi, Gunma 371-8511 Japan; 8 Higashi Sapporo Hospital, 7-35, 3-3 Higashi-Sapporo, Shiroishi-ku, Sapporo, 003-8585 Japan

**Keywords:** STXBP4, Lung squamous cell carcinoma, Drug therapy, Molecular target, Biomarker

## Abstract

**Background:**

Lung squamous cell carcinoma (LSCC) remains a challenging disease to treat, and further improvements in prognosis are dependent upon the identification of LSCC-specific therapeutic biomarkers and/or targets. We previously found that Syntaxin Binding Protein 4 (STXBP4) plays a crucial role in lesion growth and, therefore, clinical outcomes in LSCC patients through regulation of tumor protein p63 (TP63) ubiquitination.

**Methods:**

To clarify the impact of STXBP4 and TP63 for LSCC therapeutics, we assessed relevance of these proteins to outcome of 144 LSCC patients and examined whether its action pathway is distinct from those of currently used drugs in in vitro experiments including RNA-seq analysis through comparison with the other putative exploratory targets and/or markers.

**Results:**

Kaplan–Meier analysis revealed that, along with vascular endothelial growth factor receptor 2 (VEGFR2), STXBP4 expression signified a worse prognosis in LSCC patients, both in terms of overall survival (OS, *p* = 0.002) and disease-free survival (DFS, *p* = 0.041). These prognostic impacts of STXBP4 were confirmed in univariate Cox regression analysis, but not in the multivariate analysis. Whereas, TP63 (ΔNp63) closely related to OS (*p* = 0.013), and shown to be an independent prognostic factor for poor OS in the multivariate analysis (*p* = 0.0324). The action pathway of STXBP4 on suppression of TP63 (ΔNp63) was unique: Ingenuity pathway analysis using the knowledge database and our RNA-seq analysis in human LSCC cell lines indicated that 35 pathways were activated or inactivated in association with STXBP4, but the action pathway of STXBP4 was distinct from those of other current drug targets: *STXBP4, TP63* and *KDR* (VEGFR2 gene) formed a cluster independent from other target genes of tumor protein p53 (*TP53*)*,* tubulin beta 3 (*TUBB3*)*,* stathmin 1 (*STMN1*) and cluster of differentiation 274 (*CD274*: programmed cell death 1 ligand 1, PD-L1). STXBP4 itself appeared not to be a potent predictive marker of individual drug response, but we found that TP63, main action target of STXBP4, might be involved in drug resistance mechanisms of LSCC.

**Conclusion:**

STXBP4 and the action target, TP63, could afford a key to the development of precision medicine for LSCC patients.

## Background

Despite recent advances in therapeutics, lung squamous cell carcinoma (LSCC) remains a challenging disease to treat [1–4]. The advent of immune-checkpoint inhibitors along with several active target agents such as anti-angiogenic agents has altered LSCC treatment to some extent, but treatment options remain limited. The intractable patient characteristics at diagnosis; i.e., high rates of advanced stage, older age, and comorbidities, also remain a problematic issue in terms of treatment decision-making. To date, very few druggable mutations and active predictive biomarkers have been identified; thus, no LSCC-specific target therapy has yet been established. The development of precision medicine with truly active target drugs is eagerly awaited [5–9].

We recently found that Syntaxin Binding Protein 4 (STXBP4) plays a crucial role in LSCC growth through regulation of ΔNp63 (an isoform of tumor protein 63, TP63) ubiquitination and is an independent prognostic factor signifying a worse outcome in LSCC patients [10, 11]. ΔNp63 is an isoform of TP63, a member of the TP53 family, and its expression is widely used as a highly specific diagnostic marker for LSCC. ΔNp63 levels can be modulated by post-transcriptional mechanisms, mainly by ubiquitin-mediated proteolysis. Several E3 ubiquitin ligases targeting ΔNp63 have been identified so far, e.g. RACK1, NEDD4, ITCH, FBW7 and WWP1, each of them likely contributing to modulate ΔNp63 protein levels in tumors [12, 13], and we previously showed that STXBP4 binds to ΔNp63 and suppresses the anaphase-promoting complex/cyclosome (APC/C) complex-mediated proteolysis of ΔNp63, and drives the oncogenic potential of ΔNp63α [11]. STXBP4 may be a useful therapeutic target and/or marker for patients with LSCC.

These findings encouraged us to clarify the potential in clinical application of STXBP4 and its action target, TP63 (ΔNp63). In this study, we assessed whether STXBP4 and/or TP63 are truly and significantly related to patient outcome and whether STXBP4-mediated ΔNp63 degradation pathway can afford a unique therapeutic target through comparison with the other powerful prognostic biomarkers and molecular action networks of other key agents in LSCC treatment. Despite a lack of definitive prognostic markers, we selected VEGFR2 (vascular endothelial growth factor receptor 2), TUBB3 (tubulin beta 3), and PD-L1 (programmed cell death 1 ligand 1), along with p53 (tumor protein p53), ΔNp63 and STMN1 (stathmin 1), as other putative exploratory markers. Their response to drugs strongly affects the prognosis of each patient. At present, taxane, anti-angiogenesis inhibitors and immuno-checkpoint inhibitors are regarded as essential in the treatment of LSCC, the drug targets of which are TUBB3, VEGFR2, and PD-L1, respectively. Needless to say, the TP53 gene is a key factor in tumorigenesis and tumor resistance to therapy in lung cancer [5–9], and ΔNp63 is a putative diagnostic marker for LSCC [13]. STMN1 (oncoprotein 18 and LAP18) has been suggested to be a potent predictive marker for a variety of cancers including LSCC [14–17].

We further performed a genome-wide transcriptome analysis (RNA-seq) using next-generation sequencing (NGS) in 2 human LSCC cell lines, totally drug-sensitive and -resistant cells, before and after treatment with key drugs, and assessed the modulation of each exploratory target to clarify its functional molecular network.

## Methods

### Patients

Human tissue specimens were surgically resected from a total of 144 LSCC patients at Gunma University Hospital from April 2001 to December 2014. In this study, the formalin-fixed, paraffin-embedded (FFPE) tissues and clinical data obtained during the follow-up duration ranging from 4 to 164 months (median, 41 months) were used. The tumor specimens were histologically classified according to the World Health Organization criteria, and the stages were defined using the International System for Staging Lung Cancer adopted by the American Joint Committee on Cancer and the Union Internationale Centre le Cancer [18]. The study was approved by the Institutional Review Board and all patients provided written informed consent.

### Cell lines

The human LSCC cell lines, LK-2 and EBC-1 (National Institute of Biomedical Innovation/The Japanese Cancer Research Resource Bank, Osaka, Japan), NCI-H520 (American Type Culture Collection/ Summit Pharmaceuticals Intl. Corp., Tokyo, Japan), and RERF-LC-AI (Cell Engineering Division/RIKEN BioResource Research Center, Tsukuba, Ibaraki, Japan) were used. Cells were cultured in RPMI640 medium (Life Technologies, Inc., Grand Island, NY) supplemented with 10% fetal bovine serum (FBS; BioWhittaker, Verviers, Belgium). All cultured cells were incubated at 37 °C in a humidified atmosphere of 5% CO_2_ and maintained in continuous exponential growth by passaging. All cell lines were obtained from the reliable biobanks with authentication, mycoplasma test and short-tandem repeat (STR) profilings were performed in regular basis from the first culture of the cells to verify the cells to be the same as the cells registered.

### Cytotoxic analysis

Cellular sensitivity to anticancer agents was evaluated by conventional in vitro CCK8 assay following the manufacturer’s protocol (Dojindo Laboratories, Kumamoto, Japan). Exponentially growing cells (4.0 × 10^3^ cells/well) were seeded in each well of 96-microwell plates with regular medium. After incubation for 24 h, the medium was replaced, and cells were exposed to various concentrations of docetaxel (Bristol-Myers Squibb, Syracuse, NY), Cyramza/Ramucirumab (Eli Lilly-Japan, Kobe, Japan) and other cytotoxic drugs (cisplatin and 5-FU; Sigma Aldrich, Tokyo, Japan) for 72 h. Then, 10 μL of CCK-8 solution (Dojindo Laboratories, Kumamoto, Japan) was added to each well for 2 h at 37 °C, and absorbance at 450 nm was determined using an xMark Microplate Absorbance Spectrophotometer (Bio Rad, Hercules, CA, USA). From the absorbance data, the half maximal inhibitory concentration (IC_50_) was calculated with Microsoft Excel (Microsoft Corporation, Redmond, WA).

### Immuno-histochemical staining

Immuno-histochemical analysis was performed on FFPE LSCC sections. The sections were deparaffinized, blocked in protein block serum-free reagent (Dako, Carpentaria, CA) for 30 min, and incubated overnight with diluted primary antibodies at 4 °C in a humidified chamber. We used antibodies specific for STXBP4 (1:100 dilution; Abcam Japan, Tokyo, Japan), p53 (DO7, 1:50 dilution; Dako, Carpentaria, CA), TUBB3 (1:200 dilution; Abcam Japan, Tokyo, Japan) VEGFR2, STMN1 and PD-L1 (1:400 dilution, 1:400 dilution and 1:200 dilution; respectively; Cell Signaling Technology, Danvers, MA). Rabbit polyclonal ΔNp63 anti-body (1:100 dilution) was previously described [10, 11]. The reaction was visualized using the SignalStain® Boost IHC Detection Reagent (HRP, Rabbit; Cell Signaling Technology, Beverly, MA) and Histofine Simple Stain MAX-PO (Multi) Kit (Nichirei, Tokyo, Japan) according to the manufacturers’ instructions. Chromogen 3,3′-diaminobenzidine tetrahydrochloride was applied as a 0.02% solution in 50 mM ammonium acetate citric acid buffer (pH 6.0) containing 0.005% hydrogen peroxide. The sections were counterstained with Meyer’s hematoxylin (IHC World) and mounted. As negative control, the section was incubated without primary antibody to confirm its non-detectable staining [An additional file shows specificity information of each antibody and representative image of the controls (See Additional file 1)].

The expression levels of STXBP4 and ΔNp63 were scored using a semi-quantitative method: 1, ≤10%; 2, 11–25%; 3, 26–50%; 4, 51–75%; and 5, ≥76%. The percentage of STMN1 and TUBB3 staining was scored as follows: 1, ≤10%; 2, 11–25%; 3, 26–50%; and 4, ≥50%. The expression of VEGFR2 was considered positive only if distinct membrane staining was present, and was scored in the same manner as that used for STMN1 and TUBB3. For PD-L1, immunohistochemical staining was scored as 1, < 1%; 2, 1–5%; 3, 6–10%; 4, 11–25%; 5, 26–50%; and 6, > 50% of cells were positive. The tumors in which stained cancer cells were scored above 3 were defined as demonstrating high expression, with those scored 1 and 2 defined as demonstrating low expression. P53 microscopic examination of the nuclear reaction product was also undertaken and scored. P53 expression in > 10% of tumor cells was defined as positive expression. The sections were evaluated under a light microscope in a blinded fashion by at least two of the authors [An additional file shows representative image of the immune-histochemical scoring (See Additional file 2)].

### Genome-wide transcriptome analysis (RNA-seq)

Total RNA was prepared from cell lines LK-2 and RERF-LC-AI using NucleoSpin® RNA (Takara Bio Inc., Kusatsu, Shiga, Japan). The quality of the RNA was assessed by RNA integrity number (RIN) using the Agilent RNA6000 Pico Kit and the Agilent 2100 Bioanalyzer (Agilent Technologies, Santa Clara, CA, USA). High-quality RNA samples alone (RNA integrity numbers > 7.0) were used for genome-wide transcriptome analysis (RNA-seq experiments). Library preparation was performed using the TruSeq Standard mRNA Sample Prep Kit (Illumina, San Diego, CA, USA) from 1 μg of total RNA, according to the manufacturer’s protocol. The resulting libraries were subjected to paired-end sequencing using a NextSeq500 High Output v2 Kit and the Illumina NextSeq 500 system (43-base paired-end reads; Illumina). Data processing and analyses were performed using STAR v2.5.2b on the BaseSpace Sequencing Hub (Illumina). Briefly, reads were filtered, trimmed, and aligned against the UCSC human reference genome 19 (hg19) using a STAR pipeline. Normalization and differentially expressed genes were detected with TCC (Sun et al., BMC Bioinformatics, 2013) package of R software (R Foundation for Statistical Computing, Vienna, Austria. https://www.R-project.org/). Genes with a false-discovery rate (FDR)-adjusted *p*-value < 0.05 were defined as being significantly modulated genes in LK-2 and RERF-LC-A1 cells. The networks and canonical pathways were generated through the use of IPA (QIAGEN Inc., https://www.qiagenbio-informatics.com/products/ingenuity-pathway-analysis).

### Statistical analysis

Probability values (*p* value) < 0.05 indicated a statistically significant difference. The Fisher exact test was used to examine the association between two categorical variables. The correlation between drug sensitivity and gene expression value was analyzed using the parametric Pearson’s product-moment correlation analysis. The correlation among target gene modulation and other modulations was analyzed using linear regression analysis. Follow-up for the 144 patients was conducted by reference to the patient medical records. The Kaplan–Meier method was used to estimate survival as a function of time, and differences in survival were analyzed by the Cox proportional hazards model. Multivariate analyses were performed using a “survival” package in R software (Cox proportional hazards model to identify independent prognostic factors: R Foundation for Statistical Computing, Vienna, Austria. https://www.R-project.org/). Hierarchical clustering was performed by “hclust” from the stats package in R software. The day of surgery was defined as day 0 for measuring postoperative survival. OS was determined as the time from tumor resection to death from any cause. DFS was defined as the time between tumor resection and first disease progression or death. Statistical analysis was performed using R software.

## Results

### STXBP4 and patient survival

To verify its potential as therapeutic target, STXBP4 was first subjected to a comparative analysis of its clinical prognostic impact with other 6 robust targets and/or potent biomarkers used in current drug therapies: TP63 (representing ΔNp63; *TP63*), p53 (*TP53*), VEGFR2 (*KDR*), TUBB3 (*TUBB3*), STMN1 (*STMN1*) and PD-L1 (*CD274*).

A large-scale public database, The Cancer Genome Atlas (TCGA), was used to obtain data sets, for both gene expression and survival outcome, in 474 primary LSCC patients. Kaplan-Meier analysis of OS and relapse-free survival (RFS) using these data showed that *TUBB3* expression alone was correlated with RFS when patients were tentatively classified into positive- and negative-expression groups according to the expression level in each tumor (cut off set as the median, *p* = 0.001) [An additional file shows this in more details (See Additional file 3)]. Despite the lack of statistical significance, the analysis also suggested some prognostic impact of 6 molecules except *TP53*; i.e., *TP63* (*p* = 0.072), *TUBB3* (*p* = 0.091), and *STMN1* (*p* = 0.052) in OS, and *STXBP4* (*p* = 0.076), *KDR* (VEGFR2, *p* = 0.071), *STMN1* (*p* = 0.089) and *CD274* (PD-L1, *p* = 0.065) in RFS.

**Fig. 1:**
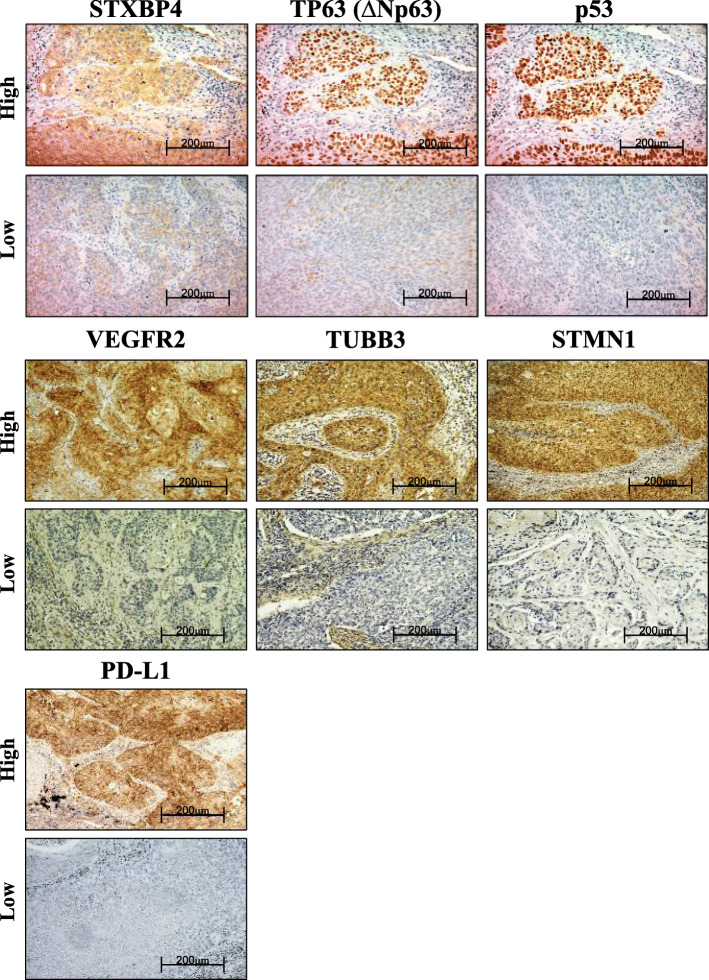
Representative immunohistochemical staining of STXBP4, TP63 (ΔNp63), p53, VEGFR2, TBB3, STMN1, and PD-L1. A total of 144 LSCC samples (formalin-fixed and paraffin-embedded sections) were stained immunohistochemically (×200, scale bar 200 μm), and classified into positive- and negative-expression groups according to the expression score evaluated by a semi-quantitative method as described in “Methods”

As a single layer of “omics” can only provide limited insights into biological significance, we performed immuno-histochemical analysis to elucidate the relevance of these 7 exploratory targets to patient outcome (Fig. 1). A total of 144 patients were enrolled in this study (Table 1). None of the patients received any cancer treatment before the operation and the majority of patients were former or current smokers (97.9%).

**Table 1:** Patient characteristics

Characteristics	No. of patients (%)
Age	
Median	72
Range	48–88
Sex	
Male	133 (92.4)
Female	11 (7.6)
Former or current smokers	
Yes	141 (97.9)
No	3 (2.1)
Pathological stage	
IA	48 (33.3)
IB	40 (27.8)
IIA	21 (14.6)
IIB	11 (7.6)
IIIA	23 (16.0)
IIIB	1 (0.70)
Recurrence	
Yes	62 (43.1)
No	82 (56.9)
Lymphatic permeation	
Yes	72 (50.0)
No	72 (50.0)
Venous Invasion	
Yes	72 (50.0)
No	72 (50.0)
Post-operative adjuvant therapy	
Yes	39 (27.1)
UFT based	17 (51.5)
TS-1 based	8 (24.2)
CBDCA based	5 (15.1)
CDDP based	3 (9.1)
No	105 (72.9)

The numbers of patients evaluated as demonstrating positive expression were 98 (68.1%) for STXBP4, 91 (63.1%) for ΔNp63 (*TP63*), 73 (50.7%) for p53, 94 (65.3%) for VEGFR2 (*KDR*), 53 (36.8%) for TUBB3, 87 (60.4%) for STMN1, and 68 (47.2%) for PD-L1 (*CD274*) [An additional file shows this in more details (See Additional file 4)]. Positivity of STXBP4 expression was not correlated with any typical clinicopathological factors including pathological stage, but closely correlated with those of ΔNp63 (*p* = 0.008) and VEGFR2 (*p* = 0.024) (See Additional file 5).

**Fig. 2:**
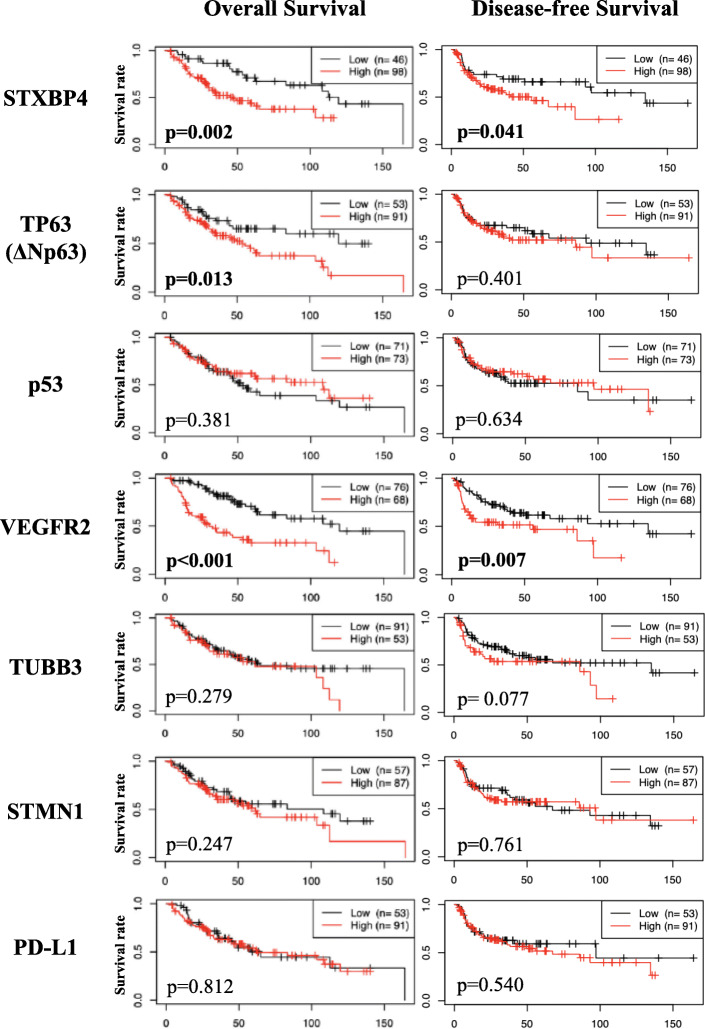
Clinical outcomes of 144 LSCC patients and expression of 7 target proteins. Kaplan-Meier analyses of overall survival (OS) and disease-free survival (DFS) were performed for 144 patients classified into high- and low-expression groups of STXBP4, TP63 (ΔNp63), p53, VEGFR2, TUBB3, STMN1, and PD-; X axis, survival time expressed in months

Kaplan–Meier analysis of OS and DFS (disease free-survival) revealed that positive STXBP4 expression signified a worse prognosis for LSCC patients, both in terms of OS (*p* = 0.002) and DFS (*p* = 0.041) (Fig. 2). Likewise, the positive expression of VEGFR2 was found to be closely connected with shorter OS (*p* < 0.001) and DFS (*p* = 0.007). The close relationship with OS was observed also for ΔNp63 (*p* = 0.013), but any other correlations with patient outcomes, both OS and DFS, were not observed for the other targets examined.

Univariate Cox regression analysis using 13 variables including 6 clinicopathological factors confirmed these observed prognostic impacts of STXBP4 (OS, *p* = 0.0021; DFS, *p* = 0.0405), TP63 (ΔNp63: OS, *p* = 0.0134) and VEGFR2 (OS, *p* < 0.001), along with several clinicopathological parameters, such as pathological stage (I/II-III) (OS, *p* = 0.0232; DFS, *p* = 0.0004), pathological T (OS, *p* = 0.0134), and lymphatic permeation (OS, *p* = 0.0267; DFS, *p* = 0.0001). Multivariate analyses revealed that the positive expression of VEGFR2 (OS, *p* < 0.0001; DFS, *p* = 0.0059) and ΔNp63 (OS, *p* = 0.0324) were independent prognostic factors for poor patient survival, together with pathological stage (DFS, *p* = 0.00096), pathologic T (OS, *p* = 0.0065) and lymphatic permeation (DFS, *p* = 0.0098), but STXBP4 was not (Table 2).

**Table 2:** Univariate and Multivariate Cox regression analysis of clinicopathological factors and protein expression levels in total patients

Clinicopathological Factors	Cox regression analysis of overall survival						Cox regression analysis of disease-free survival					
	Univariate analysis			Multivariate analysis			Univariate analysis			Multivariate analysis		
	RR	95% CI	*p*_value	RR	95% CI	*p*_value	RR	95% CI	*p*_value	RR	95% CI	*p*_value
Age (65≥/65<)	1.0869	0.56–2.08	0.8012	–	–	–	1.2365	0.60–2.50	0.5563	–	–	–
Gender (male/female)	0.7341	0.33–1.61	0.4403	–	–	–	1.2665	0.45–3.49	0.6482	–	–	–
Pathological Stage (I/II-III)	1.7621	1.08–2.87	**0.0232**	1.1665	0.64–2.10	0.6097	2.4598	1.49–4.06	**0.0004**	2.0609	1.19–3.56	**0.0096**
Pathologic T (1/2–4)	2.0430	1.15–3.60	**0.0134**	2.4608	1.28–4.70	**0.0065**	1.7041	0.98–2.95	0.0576	–	–	–
Vascular Invasion (present/absent)	0.6360	0.38–1.04	0.0722	–	–	–	0.6629	0.40–1.09	0.1104	–	–	–
Lymphatic permeation (present/absent)	1.7469	1.06–2.86	**0.0267**	1.5167	0.88–2.61	0.1331	2.7949	1.64–4.74	**0.0001**	2.1239	1.19–3.76	**0.0098**
VEGFR2 protein expression (high/low)	3.2560	1.94–5.45	**< 0.0001**	3.4920	2.01–6.05	**< 0.0001**	2.0079	1.20–3.34	**0.0073**	2.1163	1.24–3.60	**0.0059**
TUBB3 protein expression (high/low)	1.3197	0.79–2.18	0.2787	–	–	**–**	1.5876	0.95–2.65	0.0769	–	–	–
STMN1 protein expression (high/low)	1.3547	0.80–2.26	0.2473	–	–	–	1.0824	0.65–1.80	0.7605	–	–	–
STXBP4 protein expression (Pos/Neg)	2.5395	1.40–4.60	**0.0021**	1.4777	0.79–2.75	0.2186	1.8387	1.02–3.29	**0.0405**	1.4788	0.81–2.69	0.2013
∆Np63 protein expression (Pos/Neg)	2.0070	1.15–3.48	**0.0134**	1.8673	1.05–3.30	**0.0324**	1.2518	0.74–2.11	0.4007	–	–	–
p53 protein expression (Pos/Neg)	0.8030	0.49–1.31	0.3805	–	–	–	0.8854	0.53–1.46	0.6335	–	–	–
PD-L1 protein expression (Pos/Neg)	1.0638	0.63–1.77	0.8117	–	–	–	1.1789	0.69–1.99	0.5398	–	–	–

*RR* Relative risk, *CI* Confidence interval, *p* < 0.05 is considered statistically significant., calculated with continuous variables

### STXBP4 as a possible therapeutic target

**Fig. 3:**
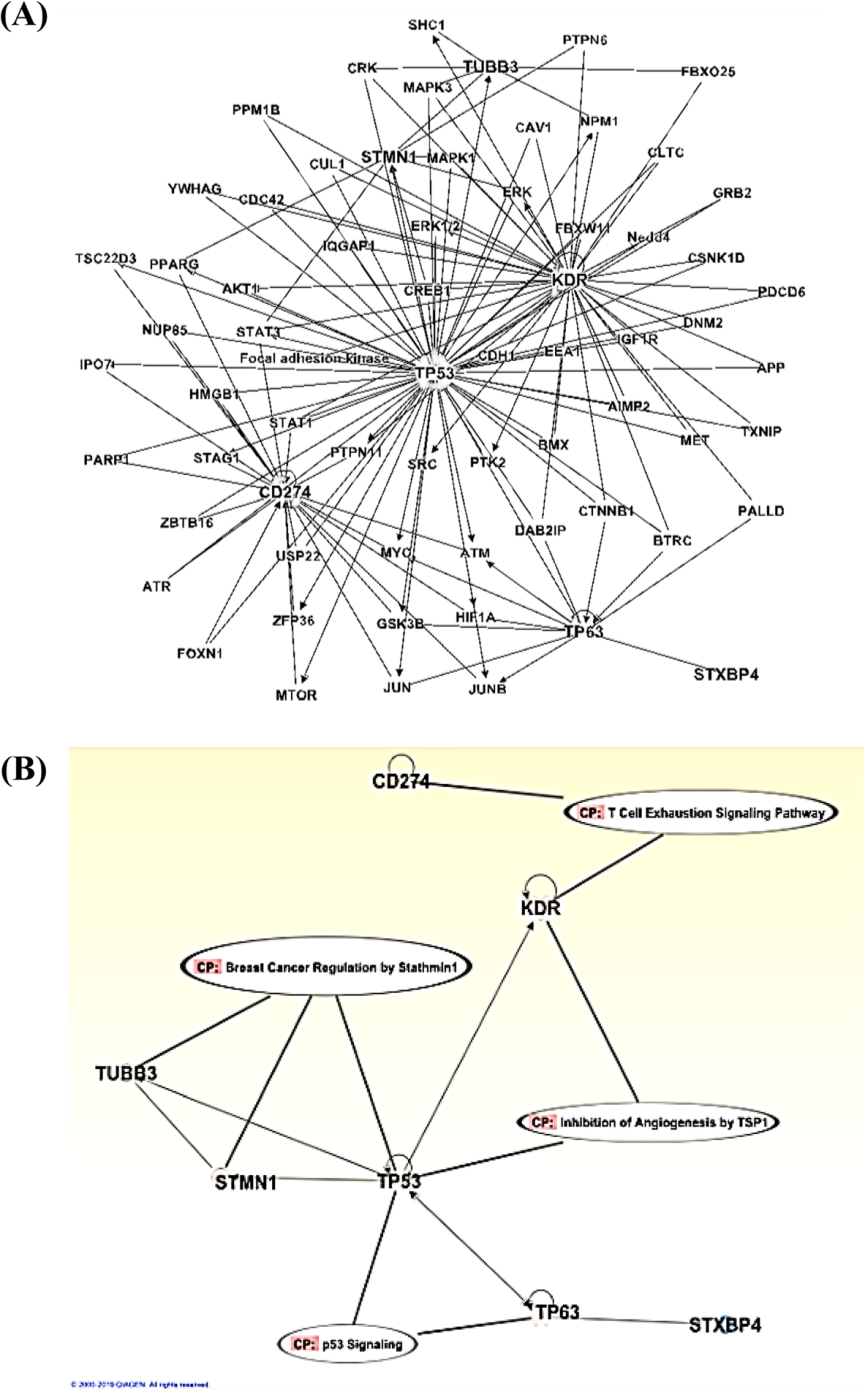
Ingenuity pathway analysis (IPA) using the knowledge database. Probable interrelations (**a**) and canonical pathways (**b**) of 7 exploratory therapeutic targets were assessed by IPA. STXBP4 acts as an up-stream regulator of TP63 (ΔNp63) and subsequently KDR (VEGFR2) via TP63, whereas the action pathway of STXBP4 was independent from those of the other 4 exploratory target genes (TP53, TUBB3, CD274 and STMN1)

The observed close relationships between VEGFR2, TUBB3, and STMN1 to patient outcome suggested the existence of some biological interactions between STXBP4 and these molecules. Ingenuity pathway analysis (IPA) using the knowledge database demonstrated that *STXBP4* acts as an up-stream regulator of *TP63* (ΔNp63) and subsequently of *KDR* (VEGFR2) via *TP63*, but the action pathway of STXBP4 was independent from those of the other 4 exploratory targets (Fig. 3a, b).

To confirm this, we performed in vitro experiments using human LSCC cell lines. According to the half-maximal inhibitory concentration (IC_50_) published on the Genomics of Drug Sensitivity in Cancer (GDSC) database (https://www.cancerrxgene.org), we first chose 4 cell lines (LK-2, EBC-1, NCI-H520, and RERF-LC-AI), and then selected 2 cell lines as totally drug-sensitive (LK-2) and -resistant cells (RERF-LC-AI). The selection was based on a CCK8 assay to confirm the cellular sensitivities to cisplatin (CDDP), 5-fluorouracil (5-FU), and docetaxel (TXT) shown on GDSC database, and newly examine their sensitivities to Ramucirumab (IC_25_); however, their cellular sensitivities to immune-check point inhibitors could not be studied using the same cytotoxic assay [An additional file shows this in more details (See Additional file 6)]. Despite the limited data, correlative analysis of drug sensitivity and gene expression (ArrayExpress, https://www.ebi.ac.uk/arrayexpress//experiments/E-MTAB-2706/) in 4 cell lines suggested that *TP63* expression was related to cellular sensitivity to CDDP [An additional file shows this in more details (See Additional file 7)].

Exposure of cells to a drug causes a dynamic alteration in gene expression, and RNA-seq analysis following such drug treatment enables us to identify all the genes modulated together in response to the drug. VEGFR2 and TUBB3 are the drug action targets of Ramucirumab and TXT, respectively, and STMN1 has been suggested to be a marker of tumor resistance to taxanes [14–17]. LK-2 and RERF-LC-AI cells were treated with or without TXT and Ramucirumab in single and combination treatment settings, and then subjected to RNA-seq analysis. We selected genes highly correlated in terms of expression level with each target gene, and then performed hierarchical clustering of canonical pathways.

**Fig. 4:**
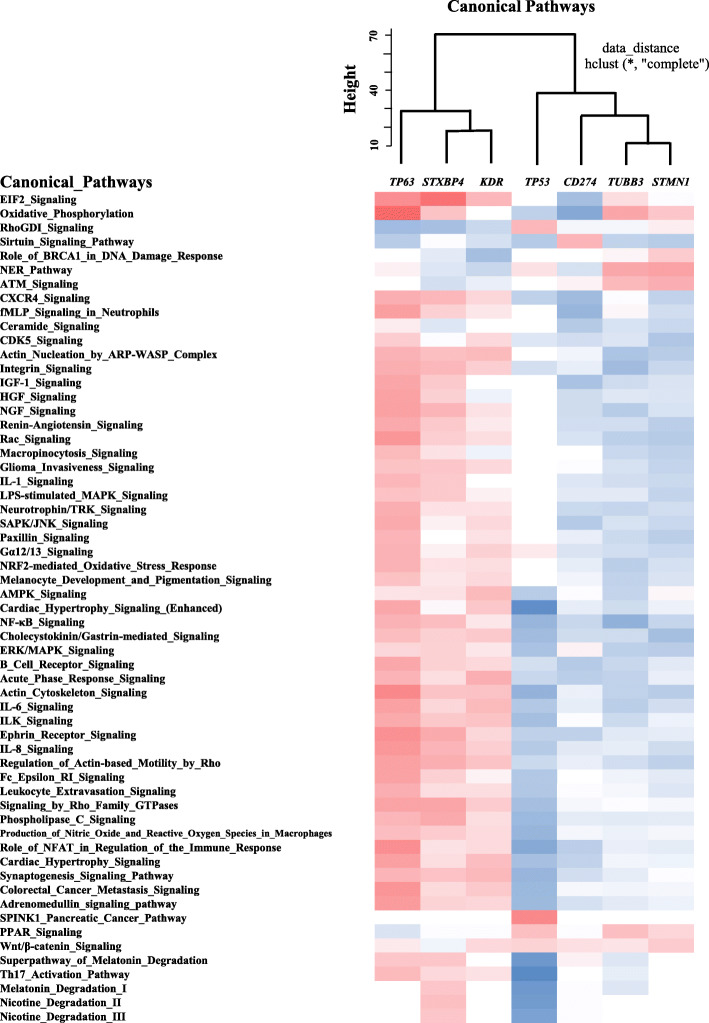
Hierarchical cluster of canonical pathways. A totally drug-sensitive LK-2 cell line and a drug-resistant RERF-LC-AI cell line were treated with or without TXT and Ramucirumab in single and combination treatment settings, and then subjected to RNA-seq analysis. Using the gene expression data, genes highly correlate in terms of expression levels of each target gene were assessed, and then hierarchical clustering of the canonical pathways was performed using “hclust” from the stats package in R software. Among the 235 target pathways, 50 representative canonical pathways are listed in this figure and the other data are shown in Additional file 5

The analysis showed that *STXBP4*, *TP63* and *KDR* (VEGFR2) formed a cluster independent from the other target genes [*TP53, TUBB3, STMN1 and CD274* (*PD-L1*)], which was in accord with the findings obtained in our previous studies (Fig. 4) [An additional file shows this in more details (See Additional file 8)] [10]. Thirty-five pathways were extracted as significantly (|activation z-score| > =2) activated or inactivated pathways in correlation with *STXBP4*. Among them, the EIF2 signaling pathway, which plays a critical role in stress-related signals to regulate both global and specific mRNA translation, was the most significantly activated [An additional file shows this in more details (See Additional file 9)].

The action pathway of STXBP4 is distinct from those of other conventional drugs such as TXT and immuno-checkpoint inhibitors. The pathway is thought to suppress 2 prominent determinants of poor prognosis in LSCC patients, TP63 and VEGFR2, and possibly p53 as well.

### STXBP4 as a possible predictive biomarker of individual therapeutic response

The observed correlations between STXBP4, ΔNp63, and VEGFR2 and clinical outcome, particularly the close correlation between STXBP4 and DFS, suggested that STXBP4 expression might afford a powerful predictive biomarker of individual response to current therapy. This hypothesis, however, cannot be directly verified due to the insufficient number of available coupled data related to clinical response and omics profiling, even when a large-scale public clinical and genomic database was used.

Our in vitro experiments clarified the relevance of each exploratory target to drug response at least in part. RNA-seq analysis revealed that *CD274* (PD-L1) expression alone was significantly higher in the totally drug-resistant RERF-LC-AI cells as the base line [An additional table file shows this in more details (See Additional file 6)]. In the drug sensitive LK-2 cells, none of the drug treatments caused any significant changes in the expression levels of the 7 targets examined (Table 3). In the resistant RERF-LC-AI cells, however, all of the drug treatments, single TXT, single Ramucirumab, and their combination, yielded a significant up-regulation in *TP63* (representing ΔNp63) and a remarkable down-regulation in *CD274.* Ramucirumab also significantly increased *STMN1* expression in the resistant cells. No changes in the expression levels of *STXBP4, KDR* (VEGFR2), or *TUBB3* were observed, regardless of the cell lines and drug treatments examined.

**Table 3:** Altered gene expression associated with exposure of cells to drugs, TXT and/or Ramucirumab

Genes	TXT			Ramucirumab			TXT + Ramucirumab		
	m_value	***p***_value	q_value	m_value	***p***_value	q_value	m_value	***p***_value	q_value
**LK-2**									
***STXBP4***	−0.0220	0.8760	1.0000	−0.0330	0.8610	1.0000	−0.0970	0.5800	1.0000
***TP63***	1.0480	0.2390	1.0000	0.7020	0.4630	1.0000	0.1620	1.0000	1.0000
***TP53***	−0.0550	0.3770	1.0000	0.0710	0.6290	1.0000	−0.0180	0.9000	1.0000
***KDR***	0.0960	0.6650	1.0000	−0.0440	0.8800	1.0000	0.2760	0.2430	1.0000
***TUBB3***	0.0530	0.4340	1.0000	0.0560	0.7100	1.0000	0.0490	0.7310	1.0000
***STMN1***	0.0670	0.2820	1.0000	0.0860	0.5560	1.0000	0.1780	0.1970	1.0000
***CD274***	0.2700	0.3117	1.0000	0.1174	0.7383	1.0000	0.3239	0.2586	1.0000
**RERF-LC-AI**									
***STXBP4***	0.2310	0.6750	1.0000	0.0840	0.9040	1.0000	−0.0240	0.9760	1.0000
***TP63***	**2.5270**	**0.0001**	**0.0270**	**1.8600**	**0.0110**	**0.5420**	**2.0460**	**0.0087**	**0.4560**
***TP53***	0.1150	0.8330	1.0000	0.0880	0.8950	1.0000	0.0850	0.9060	1.0000
***KDR***	1.3150	0.2730	1.0000	−0.2550	1.0000	1.0000	−0.1620	1.0000	1.0000
***TUBB3***	0.2040	0.6940	1.0000	0.8610	0.1720	1.0000	0.7020	0.3030	1.0000
***STMN1***	0.0420	0.9350	1.0000	**1.6410**	**0.0104**	**0.5330**	1.2510	0.0690	1.0000
***CD274***	**−1.3328**	**0.0116**	**0.5476**	**−1.5704**	**0.0144**	**0.6156**	**−1.5697**	**0.0240**	**0.7406**

m_value, log2 fold change (with and without treatment); q_value, false discovery rate (FDR)

These findings suggested that the high-level expression of *CD274* (PD-L1) is related to cellular drug resistance, at least in part, but could be partially down-regulated by TXT and/or Ramucirumab. *TP63* (ΔNp63) induction might be involved in the cellular resistance mechanisms of LSCC to TXT and/or Ramucirumab treatment, and the up-regulation of STMN1 could also participate in Ramucirumab resistance. These findings may afford some help in the development of precision medicine for LSCC patients, with the optimal treatment for individual LSCC patients selected through expression analysis of *CD274, TP63*, and *STMN1*. STXBP4 is a potent prognostic marker in LSCC patients but not a powerful predictive marker of individual response to widely used current therapeutic drugs.

## Discussion

Despite the advent of new treatment options, advanced and metastatic LSCCs remain difficult-to-treat malignancies. Extensive work is underway to expand the treatment options. Among the work in progress, druggable targets specific to the disease and biomarkers for optimal treatment selection have been intensively researched to develop precision medicine with truly active target drugs [1–9, 19]. We have been involved in these researches and identified STXBP4 as a possible therapeutic target in LSCC by elucidating its biological function in the malignancy [9, 10].

In this study, we demonstrated that STXBP4-mediated TP63 (ΔNp63) modulation pathway may play an important role in survival outcome of LSCC patient, and first suggested that *TP63* (ΔNp63) induction might be involved in the cellular resistance mechanisms of the widely used current key drugs CDDP, TXT, and Ramucirumab. ΔNp63 is a putative diagnostic marker for LSCC [13], and would be a potent predictive biomarker of therapeutic resistance to current standard drug-therapy. STXBP4 act as an up-stream regulator of TP63 (ΔNp63), and drives the oncogenic potential of ΔNp63α [10, 11]. Reduction in *TP63* (ΔNp63) expression by STXBP4 might ameliorate tumor resistance to the current drug treatments and prolong survival of LSCC patient. Interestingly, IPA indicated that the action pathway of *STXBP4* was independent from those of the other 4 targets examined in this study, with *STXBP4, TP63* and *KDR* (VEGFR2) found to form a cluster independent from the other genes, *TP53, TUBB3, STMN1* and *CD274* (PD-L1), suggesting STXBP4 possibly to be a novel therapeutic target. STXBP4 and the action target, TP63, could afford a key to the development of precision medicine for LSCC patients.

The prognostic impact of STXBP4 and TP63 (ΔNp63) expression, however, still needs to be evaluated by continuous studies. There observed some discrepant results between our previous cohort study (87 patients) and this scale-up cohort study (144 patients) [10]: Current study indicated that STXBP4 was not an independent prognostic factor of both OS and PFS, and did not relate to any clinicopathological parameters including pathological stages. Even so, Kaplan-Meier analysis and univariate COX regression analysis in 144 LSCC patients showed that positive STXBP4 expression signified a worse prognosis for LSCC patients, and TP63 (ΔNp63), an action target of STXBP4, was evaluated to be an independent prognostic factor for poor OS. Since there was no significant difference in patient background and STXBP4-positvity evaluation process, this might be due to the scale-bias of 2 studies. Despite of a small difference in the statistical evaluation, we may conclude that STXBP4 and TP63 (ΔNp63) play an important role in survival in LSCC patients, at least in a part.

This study further demonstrated that the action pathway of STXBP4 differs from those of current key agents. Among 35 pathways activated or inactivated is association with *STXBP4*, the EIF2 signaling pathway was the most significantly activated. eIF2β, a subunit of the heterotrimeric G protein EIF2 that functions as a transcription initiation factor, was recently reported to play a critical role in stress-related signals to regulate both global and specific mRNA translation, and is highly up-regulated in lung cancer specimens on multi-omics levels (DNA, RNA, and protein) [20]. All the details are still not definitive [12, 13], but we previously showed that the inhibition of STXBP4 results in the suppression of TP63 (ΔNp63), a p53 family protein, and inhibits tumor growth [10, 11]. Despite additional researches were indispensable, STXBP4 could represent an unprecedented and unique therapeutic target to improve LSCC treatment.

Our in vitro data also would lead some contribution in the progress in development research on LSCC precision medicine. We found here that TP63 (ΔNp63) induction might be involved in the cellular resistance mechanisms of LSCC to CDDP, TXT and Ramucirumab therapies. Despite the limited in vitro data, our RNA-seq analysis also showed that the high-level expression of *CD274* (PD-L1) might be related to cellular drug resistance in LSCCs. The increased *CD274* expression could be partially down-regulated by TXT and/or Ramucirumab treatments, but the up-regulation of STMN1 possibly participates in resistance to TXT and Ramucirumab. Among all the findings, down-regulation of CD274 caused by TXT and Ramucirumab in RELF-LC-A1 cells was unexpected results, because cellular stress response signals (i.e eIF2, NFkb, mTOR and OXPHOS) modulated by chemotherapeutic drugs are known to mostly up-regulate PD-L1 expression in cancer cells [21–23]. Whereas, we can find several contradictory findings that STAT3 silencing, several non-coding RNAs (i.e NKX2–1-AS1 and miR-197), and microtubule targeting agent down-regulates *CD274* at transcriptional level [24–26]. It has been also shown that TP63 regulates various miRNAs that affects multiple targets gene transcription [27], and several miRNAs relate to over expression of *CD274* (miR-3127-5p, miR135, miR-20b, miR-21, miR-130b) as well as down regulation of *CD274* (miRNA142-5p and miR197) [22, 28]. Together with our findings that TP63 (ΔNp63) induction might be involved in the cellular resistance mechanisms and VEGFR2 expression closely related to that of TP63, these could support the observation that TXT and Ramucirumab treatment could down-regulate CD274.

Despite the limited data, our data suggest the potential that the optimal treatment for individual LSCC patients could be selected through expression analysis of *CD274* (for immune-checkpoint inhibitor), *STMN1* (for TXT and Ramucirumab), and *TP63* (for all treatment failures including platinum agents). Along with that of STXBP4 (*p* = 0.041), the expression levels of VEGFR2 (*p* = 0.007) and TUBB3 (*p* = 0.077) were closely and potentially connected with DFS. For the selection of TXT and Ramucirumab, additional expression analysis of each target molecule; *TUBB3* and *KDR* (VEGFR2), respectively, would be helpful.

In LSCC, none of the powerful predictive marker of individual response has been established yet. High expression of CD274 might be a selection marker of PD-L1 inhibitor, while high STMN1 expression would be a possible marker to avoid TXT and Ramucirumab treatment. Likewise, high expression of TUBB3 and KDR (VEGFR2), could be a selection marker respectively for TXT and Ramucirumab treatment, and high TP63 could be a multidrug resistant marker. Needless to say, further intensive studies are strongly required to probe the clinical utility, these findings may afford some help in the development of precision medicine for LSCC patients.

These findings are partially validated in clinical practice, although definitive predictive markers for CDDP, taxane, antiangiogenetic inhibitors and immune-checkpoint inhibitors remain controversial. The use of the immune-checkpoint inhibitor (Pembrolizumab) for metastatic LSCC patients with tumors showing 50% or greater PD-L1 (*CD274*) is now widely recognized as a standard first-line therapy [1–3, 29]. The putative predictive markers of Ramucirumab-based regimens remain unclear [21], but high-level STMN1 expression was demonstrated as a potent determinant of chemo-resistance and, thus, a poor prognosis in LSCC patients [14]. The AKT/FOXM1/STMN1 pathway was indicated to drive resistance to tyrosine kinase inhibitors in advanced non-small cell lung cancer including LSCC [30]. TP63 is an action target of STXBP4 [10, 11, 13]. The development of STXBP4 inhibitors is considered to be key to the development of precision medicine with truly active target drugs for LSCC patients, although the detailed impact of STXBP4 and TP63 (ΔNp63) for patient outcome, tumor control and drug sensitivity, and its possibility to be a druggable target still needs to be validated. In parallel with the basic researches to elucidate their detailed biological functions and active inhibitors, a larger-scale cohort study is now in progress.

## Conclusions

Herein, we demonstrated that STXBP4 and TP63 (ΔNp63) could afford unprecedented and unique therapeutic seeds to improve LSCC treatment. The development of STXBP4 inhibitors would not only expand treatment options but also lead to precision medicine guided by expression analysis of several key genes such as *CD274* (for immune-checkpoint inhibitor), *STMN1* (for TXT and Ramucirumab), and *TP63* (for all treatment failures including platinum agents) in LSCC patients.

## Supplementary information

**Additional file 1.** Antibody specificity information and representative images of immunohistochemical staining (p53) including Hematoxylin Eosin staining and negative control.

**Additional file 2.** Representative images of immunohistochemical scoring. (A)STXBP4 and ΔNp63 were scored from 1 to 5; (B) PD-L1 staining were scored from 1 to 6; (C) VEGFR2, TUBB3 and STMN1 were scored from 1 to 4. All images were shown in × 200 magnification. Scale bars, 200 μm.

**Additional file 3.** Clinical outcomes of 474 LSCC patients and expression of 7 target genes, STXBP4, TP63 (ΔNp63), TP53 (p53), VEGFR2, TUBB3, STMN1, and CD274 (PD-L1). Analysis using available data sets of 474 primary LSCC patients in a large-scale public database, The Cancer Genome Atlas (TCGA). Kaplan-Meier analyses of overall survival (OS) and relapse-free survival (RFS) were performed for all patients after classification into high- and low-expression groups with the median expression level of each gene used as the cut off value; X axis, survival time expressed in days.

**Additional file 4.** Expression Score: Immunohistochemical staining of target protein in 144 LSCC patients

**Additional file 5.** STXBP4 expression and clinicopathological factors

**Additional file 6.** In vitro data released in public databases. (A) Cellular sensitivity to 4 key drugs in the “Genomics of Drug Sensitivity in Cancer” database; (B) Expression of 7 genes (RNA-seq data) in the “ArrayExpress” database

**Additional file 7.** Expression levels of genes correlated with cellular sensitivity to 4 key drugs.

**Additional file 8.** Hierarchical cluster of canonical pathways. Following Fig. 2, the data for the remaining 185 canonical pathways are shown in this figure.

**Additional file 9.** Thirty-five canonical pathways significantly modulated (activated or inactivated) (z-score ≥ 2) by TXT and/or Ramucirumub treatment. A totally drug-sensitive LK-2 cell line and a drug -resistant RERF-LC-AI cell line were treated with or without TXT and Ramucirumab in single and combination treatment settings, and then subjected to RNA-seq analysis. Using the gene expression data, genes highly correlated in terms of expression level with each target gene were assessed, and the 35 most significantly modulated (activated or inactivated) canonical pathways were identified.

## Data Availability

The data of this study were derived from the The Cancer Genome Atlas (TCGA) and ArrayExpress, which were available respectively from https://www.cancer.gov/about-nci/organization/ccg/research/structural-genomics/tcga and https://www.ebi.ac.uk/arrayexpress//experiments/E-MTAB-2706/. The datasets used and analysed during the current study are available from the corresponding author on reasonable request.

## Abbreviations

LSCC: Lung squamous cell carcinoma
STXBP4: Syntaxin Binding Protein 4
TP63: Tumor protein p63
ΔNp63: An isoform of TP63
VEGFR2: Vascular endothelial growth factor receptor 2
OS: Overall survival
DFS: Disease-free survival
TP53: Tumor protein p53
TUBB3: Tubulin beta 3
STMN1: Stathmin 1
CD274: Cluster of differentiation 274 (PD-L1, programmed cell death 1 ligand 1)
NGS: Next-generation sequencing
RFS: Relapse-free survival
