# Supplementary material for: Distinctive roles of syntaxin binding protein 4 and its action target, TP63, in lung squamous cell carcinoma: a theranostic study for the precision medicine
Source: BMC Cancer. 2020 Sep 29;20:935. doi: 10.1186/s12885-020-07448-2 (PMC7526255; doi:10.1186/s12885-020-07448-2)
Supplement: Supplementary file 1 — Additional file 1. Antibody specificity information and representative images of immunohistochemical staining (p53) including Hematoxylin Eosin staining and negative control. [file 12885_2020_7448_MOESM1_ESM.pptx]

## Slide 1
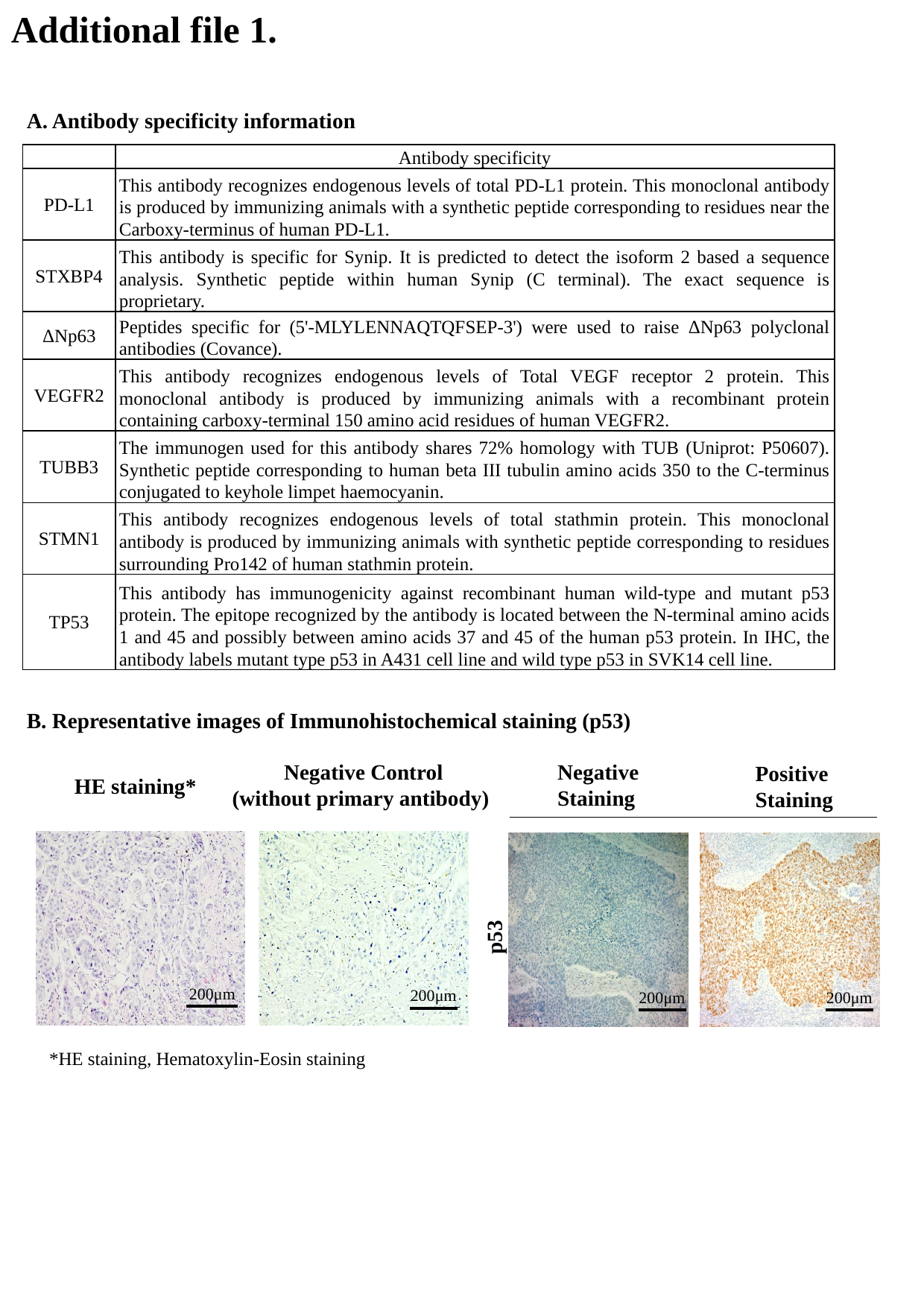

Additional file 1.
A. Antibody specificity information
| | Antibody specificity |
| --- | --- |
| PD-L1 | This antibody recognizes endogenous levels of total PD-L1 protein. This monoclonal antibody is produced by immunizing animals with a synthetic peptide corresponding to residues near the Carboxy-terminus of human PD-L1. |
| STXBP4 | This antibody is specific for Synip. It is predicted to detect the isoform 2 based a sequence analysis. Synthetic peptide within human Synip (C terminal). The exact sequence is proprietary. |
| ΔNp63 | Peptides specific for (5'-MLYLENNAQTQFSEP-3') were used to raise ΔNp63 polyclonal antibodies (Covance). |
| VEGFR2 | This antibody recognizes endogenous levels of Total VEGF receptor 2 protein. This monoclonal antibody is produced by immunizing animals with a recombinant protein containing carboxy-terminal 150 amino acid residues of human VEGFR2. |
| TUBB3 | The immunogen used for this antibody shares 72% homology with TUB (Uniprot: P50607). Synthetic peptide corresponding to human beta III tubulin amino acids 350 to the C-terminus conjugated to keyhole limpet haemocyanin. |
| STMN1 | This antibody recognizes endogenous levels of total stathmin protein. This monoclonal antibody is produced by immunizing animals with synthetic peptide corresponding to residues surrounding Pro142 of human stathmin protein. |
| TP53 | This antibody has immunogenicity against recombinant human wild-type and mutant p53 protein. The epitope recognized by the antibody is located between the N-terminal amino acids 1 and 45 and possibly between amino acids 37 and 45 of the human p53 protein. In IHC, the antibody labels mutant type p53 in A431 cell line and wild type p53 in SVK14 cell line. |
B. Representative images of Immunohistochemical staining (p53)
Negative Control
(without primary antibody)
HE staining*
200μm
200μm
Negative
Staining
Positive
Staining
p53
200μm
200μm
*HE staining, Hematoxylin-Eosin staining
