# Supplementary material for: Distinctive roles of syntaxin binding protein 4 and its action target, TP63, in lung squamous cell carcinoma: a theranostic study for the precision medicine
Source: BMC Cancer. 2020 Sep 29;20:935. doi: 10.1186/s12885-020-07448-2 (PMC7526255; doi:10.1186/s12885-020-07448-2)
Supplement: Supplementary file 2 — Additional file 2. Representative images of immunohistochemical scoring. (A)STXBP4 and ΔNp63 were scored from 1 to 5; (B) PD-L1 staining were scored from 1 to 6; (C) VEGFR2, TUBB3 and STMN1 were scored from 1 to 4. All images were shown in × 200 magnification. Scale bars, 200 μm. [file 12885_2020_7448_MOESM2_ESM.pptx]

## Slide 1
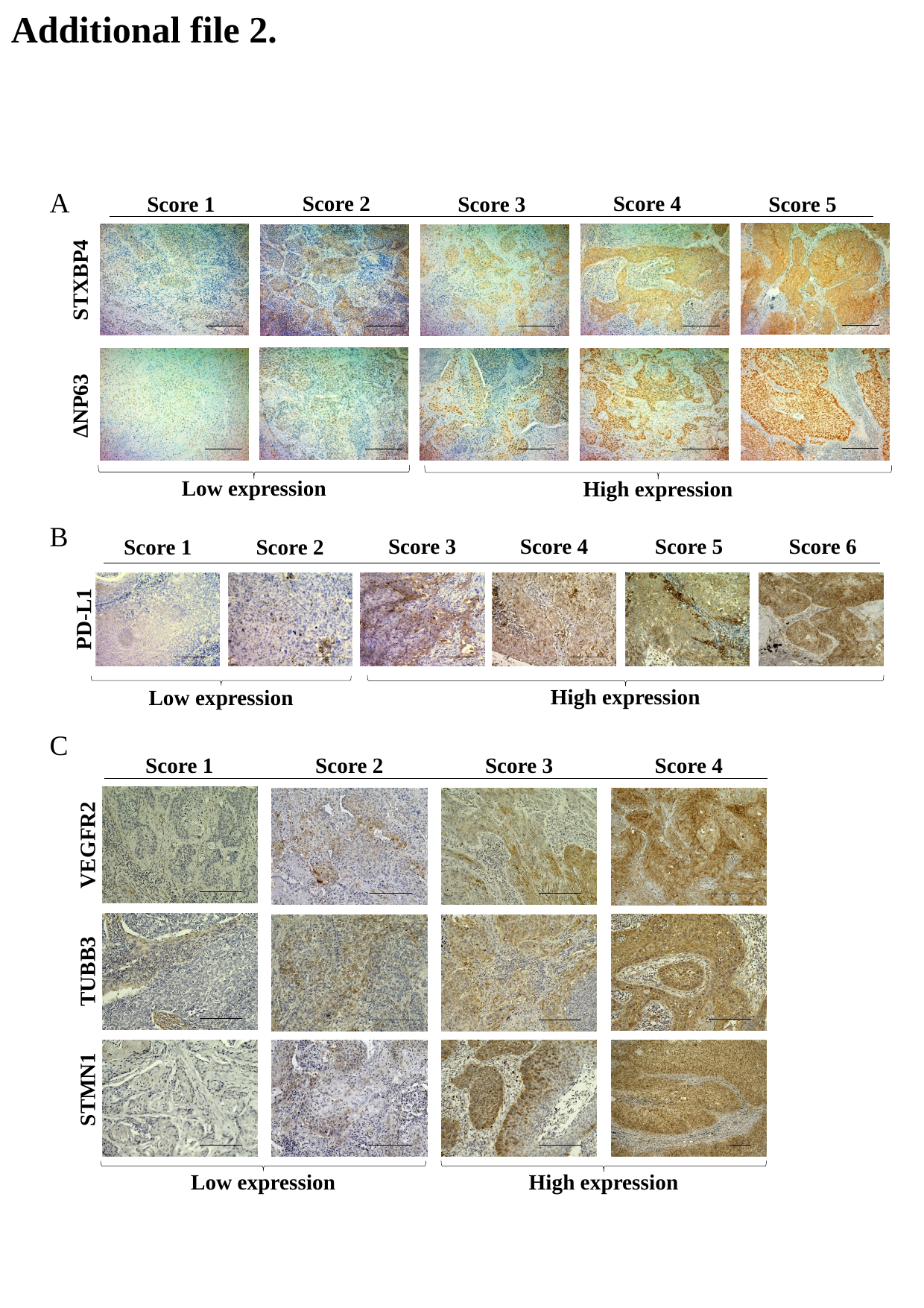

Additional file 2.
A
Score 2
Score 4
Score 1
Score 3
Score 5
STXBP4
∆NP63
Low expression
High expression
B
Score 3
Score 4
Score 5
Score 6
Score 1
Score 2
PD-L1
High expression
Low expression
C
Score 2
Score 1
Score 3
Score 4
VEGFR2
TUBB3
STMN1
Low expression
High expression
