# Supplementary material for: Distinctive roles of syntaxin binding protein 4 and its action target, TP63, in lung squamous cell carcinoma: a theranostic study for the precision medicine
Source: BMC Cancer. 2020 Sep 29;20:935. doi: 10.1186/s12885-020-07448-2 (PMC7526255; doi:10.1186/s12885-020-07448-2)
Supplement: Supplementary file 3 — Additional file 3. Clinical outcomes of 474 LSCC patients and expression of 7 target genes, STXBP4, TP63 (ΔNp63), TP53 (p53), VEGFR2, TUBB3, STMN1, and CD274 (PD-L1). Analysis using available data sets of 474 primary LSCC patients in a large-scale public database, The Cancer Genome Atlas (TCGA). Kaplan-Meier analyses of overall survival (OS) and relapse-free survival (RFS) were performed for all patients after classification into high- and low-expression groups with the median expression level of each gene used as the cut off value; X axis, survival time expressed in days. [file 12885_2020_7448_MOESM3_ESM.pptx]

## Slide 1
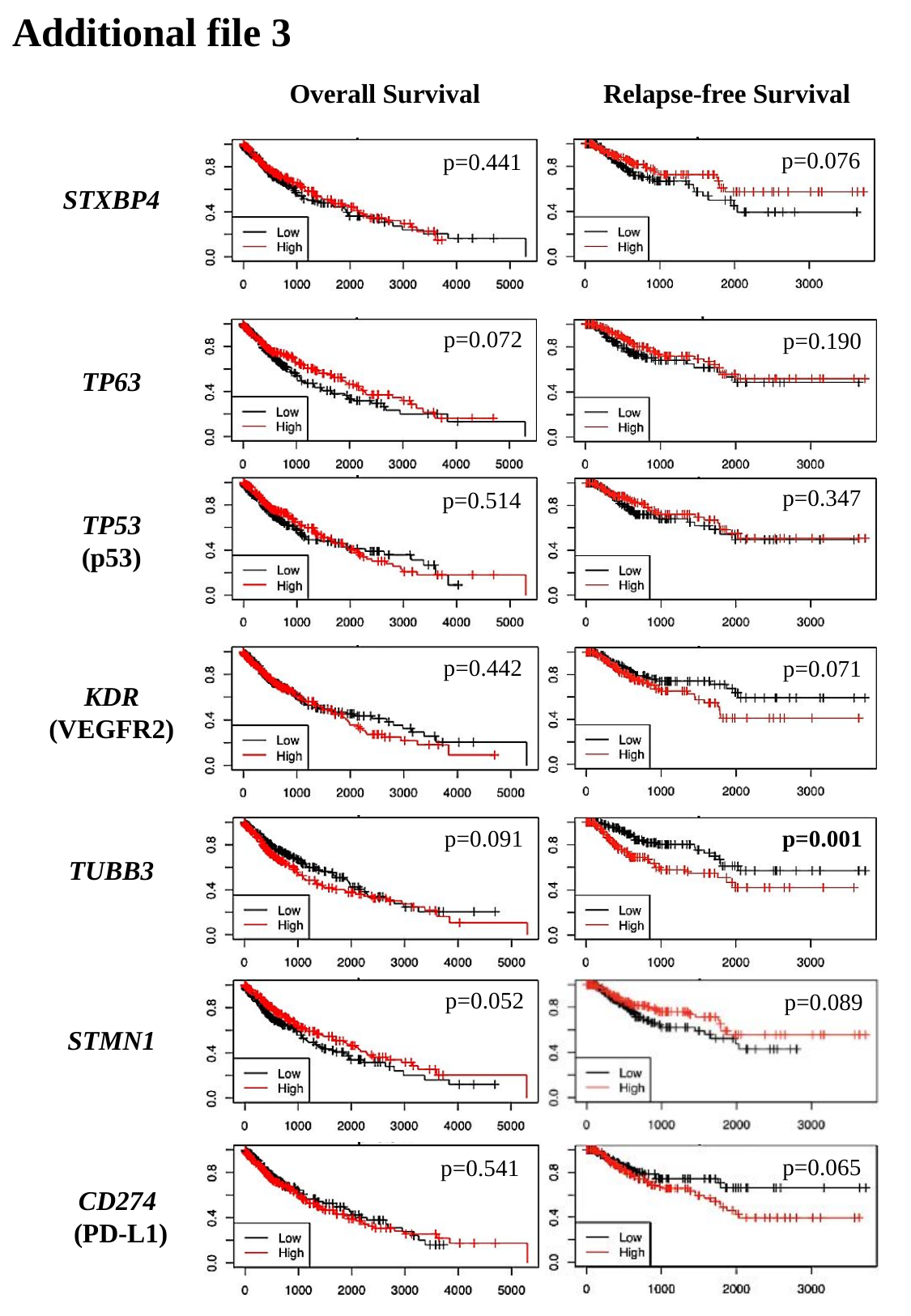

Additional file 3
Overall Survival
Relapse-free Survival
p=0.076
p=0.441
STXBP4
p=0.072
p=0.190
TP63
p=0.347
p=0.514
TP53
(p53)
p=0.442
p=0.071
KDR
(VEGFR2)
p=0.091
p=0.001
TUBB3
p=0.052
p=0.089
STMN1
p=0.065
p=0.541
CD274
(PD-L1)
