# Supplementary material for: Distinctive roles of syntaxin binding protein 4 and its action target, TP63, in lung squamous cell carcinoma: a theranostic study for the precision medicine
Source: BMC Cancer. 2020 Sep 29;20:935. doi: 10.1186/s12885-020-07448-2 (PMC7526255; doi:10.1186/s12885-020-07448-2)
Supplement: Supplementary file 4 — Additional file 4. Expression Score: Immunohistochemical staining of target protein in 144 LSCC patients [file 12885_2020_7448_MOESM4_ESM.docx]

**Additional file 4. Expression Score: Immunohistochemical staining of target protein in 144 SCLC patients**

| **Target protein** | **Expression Level [n (%)]** | | | | | |
| --- | --- | --- | --- | --- | --- | --- |
|  | **Score 1** | **Score 2** | **Score 3** | **Score 4** | **Score 5** | **Score 6** |
| STXBP4 | 17 (11.8) | 29 (20.1) | 12 (8.33) | 35 (24.3) | 51 (35.4) | - |
| ΔNp63 | 15 (10.4) | 38 (26.4) | 18 (12.5) | 39 (27.1) | 34 (23.6) | - |
| VEGFR2 | 50 (34.7) | 26 (18.1) | 53 (36.8) | 15 (10.4) | - | - |
| TUBB3 | 52 (36.1) | 39 (27.1) | 28 (19.4) | 25 (17.4) | - | - |
| STMN1 | 30 (20.8) | 27 (18.8) | 24 (16.7) | 63 (43.8) | - | - |
| PD-L1 | 32 (22.2) | 21 (14.6) | 23 (16.0) | 26 (18.1) | 18 (12.5) | 24 (16.7) |
|  | **Negative** | | **Positive** | | | |
| TP53 | 71 (49.3) | | 73 (50.7) | | | |

Blue column was evaluated as positive expression.
