# Supplementary material for: Distinctive roles of syntaxin binding protein 4 and its action target, TP63, in lung squamous cell carcinoma: a theranostic study for the precision medicine
Source: BMC Cancer. 2020 Sep 29;20:935. doi: 10.1186/s12885-020-07448-2 (PMC7526255; doi:10.1186/s12885-020-07448-2)
Supplement: Supplementary file 5 — Additional file 5. STXBP4 expression and clinicopathological factors [file 12885_2020_7448_MOESM5_ESM.docx]

**Additional file 5. STXBP4 expression and clinicopathological factors.**

|  |  | STXBP4 Expression [n(%)] | | |
| --- | --- | --- | --- | --- |
| Characteristics | | Low | High | p. value |
| Age | Median | 72 | 73 |  |
|  | Range | 59-86 | 48-89 |  |
|  |  |  |  |  |
| Sex | Male | 42 (29.2) | 91 (63.2) | 0.7436 |
|  | Female | 4 (2.8) | 7 (4.8) |  |
|  |  |  |  |  |
| Former or current smokers | Yes | 43 (30.3) | 96 (67.6) | 0.9292 |
|  | No | 1 (0.7) | 2 (1.4) |  |
|  |  |  |  |  |
| Pathological stage | IA | 18 (12.5) | 30 (20.8) | 0.6128 |
|  | IB | 15 (10.4) | 25 (17.3) |  |
|  | IIA | 5 (3.5) | 16 (11.1) |  |
|  | IIB | 3 (2.1) | 8 (5.6) |  |
|  | IIIA | 5 (3.5) | 18 (12.5) |  |
|  | IIIB | 1 (0.7) | 0 (0) |  |
|  |  |  |  |  |
| Recurrence | Yes | 28 (19.4) | 54 (37.5) | 0.5146 |
|  | No | 18 (12.5) | 44 (30.6) |  |
|  |  |  |  |  |
| Lymphatic permeation | Yes | 20 (13.9) | 52 (36.1) | 0.2836 |
|  | No | 26 (18.1) | 46 (31.9) |  |
|  |  |  |  |  |
| Venous Invasion | Yes | 20 (13.9) | 52 (36.1) | 0.2836 |
|  | No | 26 (18.1) | 46 (31.9) |  |
|  |  |  |  |  |
| ΔNp63 | High | 20 (13.8) | 71 (49.3) | **0.0008** |
|  | Low | 26 (18.1) | 27 (18.8) |  |
|  |  |  |  |  |
| VEGFR2 | High | 24 (16.7) | 70 (48.6) | **0.0236** |
|  | Low | 22 (15.3) | 28 (19.4) |  |
|  |  |  |  |  |
| TUBB3 | High | 13 (9.0) | 40 (27.8) | 0.1452 |
|  | Low | 33 (22.9) | 58 (40.3) |  |
|  |  |  |  |  |
| STMN1 | High | 23 (16.0) | 64 (44.4) | 0.0799 |
|  | Low | 23 (16.0) | 34 (23.6) |  |
|  |  |  |  |  |
| PD-L1 | High | 29 (20.1) | 62 (43.1) | 0.9795 |
|  | Low | 17 (11.8) | 36 (25.0) |  |
|  |  |  |  |  |
| P53 | Positive | 23 (16.0) |  |  |
|  | Negative | 23 (16.0) |  |  |
