# Supplementary material for: Distinctive roles of syntaxin binding protein 4 and its action target, TP63, in lung squamous cell carcinoma: a theranostic study for the precision medicine
Source: BMC Cancer. 2020 Sep 29;20:935. doi: 10.1186/s12885-020-07448-2 (PMC7526255; doi:10.1186/s12885-020-07448-2)
Supplement: Supplementary file 6 — Additional file 6. In vitro data released in public databases. (A) Cellular sensitivity to 4 key drugs in the “Genomics of Drug Sensitivity in Cancer” database; (B) Expression of 7 genes (RNA-seq data) in the “ArrayExpress” database [file 12885_2020_7448_MOESM6_ESM.docx]

**Additional file 6. *In vitro* data released in public databases**

1. **Cellular sensitivity to 4 key drugs in the “Genomics of Drug Sensitivity in Cancer” database**

|  | IC_50_ (uM) | | | |
| --- | --- | --- | --- | --- |
|  | LK-2 | EBC-1 | NCI-H520 | RERF-LC-AI |
| TXT | 0.00184 | 0.00147 | 0.0038 | 0.00909 |
| CDDP | 27.9 | 29.0 | 28.1 | 92.1 |
| 5-FU | 6.5 | 230 | 3.6 | 93.6 |
| Ramucirumab* | 6.6 | 6.8 | 7.0 | 12.8 |

*, IC_25_ values obtained by our experiments (72h CCK-8 assay)

1. **Expression of 7 genes (RNA-seq data) in the “ArrayExpress” database**

|  | RPKM | | | |  |
| --- | --- | --- | --- | --- | --- |
|  | LK-2 | EBC-1 | NCI-H520 | RERF-LC-AI |  |
| *STXBP4* (RPKM) | 1.83 | 1.95 | 2.83 | 0.89 |  |
| *KDR* (RPKM) | 0.51 | 0.00 | 0.33 | 0.00 |  |
| *TP63 (RPKM)* | 0.04 | 0.02 | 0.00 | 0.12 |  |
| *TP53 (RPKM)* | 60.65 | 1.49 | 2.02 | 4.47 |  |
| *STMN1* (RPKM) | 109.69 | 115.48 | 53.94 | 117.77 |  |
| *CD274* (RPKM) | **0.53** | 6.27 | 0.22 | **16.87** |  |

RPKM, Reads per kilobase of exon per million mapped reads
