# Supplementary material for: Distinctive roles of syntaxin binding protein 4 and its action target, TP63, in lung squamous cell carcinoma: a theranostic study for the precision medicine
Source: BMC Cancer. 2020 Sep 29;20:935. doi: 10.1186/s12885-020-07448-2 (PMC7526255; doi:10.1186/s12885-020-07448-2)
Supplement: Supplementary file 7 — Additional file 7. Expression levels of genes correlated with cellular sensitivity to 4 key drugs. [file 12885_2020_7448_MOESM7_ESM.docx]

**Additional file 7. Expression levels of genes correlated with cellular sensitivity to 4 key drugs**

|  |  | **TXT** | |  | **CDDP** | |  | **5-FU** | |  | **Ramucirumab** | |
| --- | --- | --- | --- | --- | --- | --- | --- | --- | --- | --- | --- | --- |
| **Gene symbol** |  | rho_value | p_value |  | rho_value | p_value |  | rho_value | p_value |  | rho_value | p_value |
| ***STXBP4*** |  | -0.6351 | 0.3649 |  | -0.8303 | 0.1697 |  | -0.2809 | 0.7191 |  | -0.8011 | 0.1989 |
| ***KDR*** |  | -0.4536 | 0.5464 |  | -0.5639 | 0.4361 |  | -0.8106 | 0.1894 |  | -0.5730 | 0.4270 |
| ***TP63*** |  | 0.8517 | 0.1483 |  | 0.9588 | **0.0412** |  | 0.0755 | 0.9245 |  | 0.9428 | 0.0572 |
| ***TP53*** |  | -0.3788 | 0.6212 |  | -0.3006 | 0.6994 |  | -0.4933 | 0.5067 |  | -0.3395 | 0.6605 |
| ***TUBB3*** |  | -0.3466 | 0.6534 |  | -0.1046 | 0.8954 |  | 0.1273 | 0.8727 |  | -0.1563 | 0.8437 |
| ***STMN1*** |  | 0.1241 | 0.8759 |  | 0.4129 | 0.5871 |  | 0.5556 | 0.4444 |  | 0.3699 | 0.6301 |
| ***CD274*** |  | 0.8249 | 0.1751 |  | 0.9390 | 0.0610 |  | 0.4162 | 0.5838 |  | 0.9344 | 0.0656 |

RNA-seq data from “ArrayExpress” and drug sensitivity data from “Genomics of Drug Sensitivity in Cancer” were used for the analysis
