# Supplementary material for: Distinctive roles of syntaxin binding protein 4 and its action target, TP63, in lung squamous cell carcinoma: a theranostic study for the precision medicine
Source: BMC Cancer. 2020 Sep 29;20:935. doi: 10.1186/s12885-020-07448-2 (PMC7526255; doi:10.1186/s12885-020-07448-2)
Supplement: Supplementary file 8 — Additional file 8. Hierarchical cluster of canonical pathways. Following Fig. 2, the data for the remaining 185 canonical pathways are shown in this figure. [file 12885_2020_7448_MOESM8_ESM.pptx]

## Slide 1
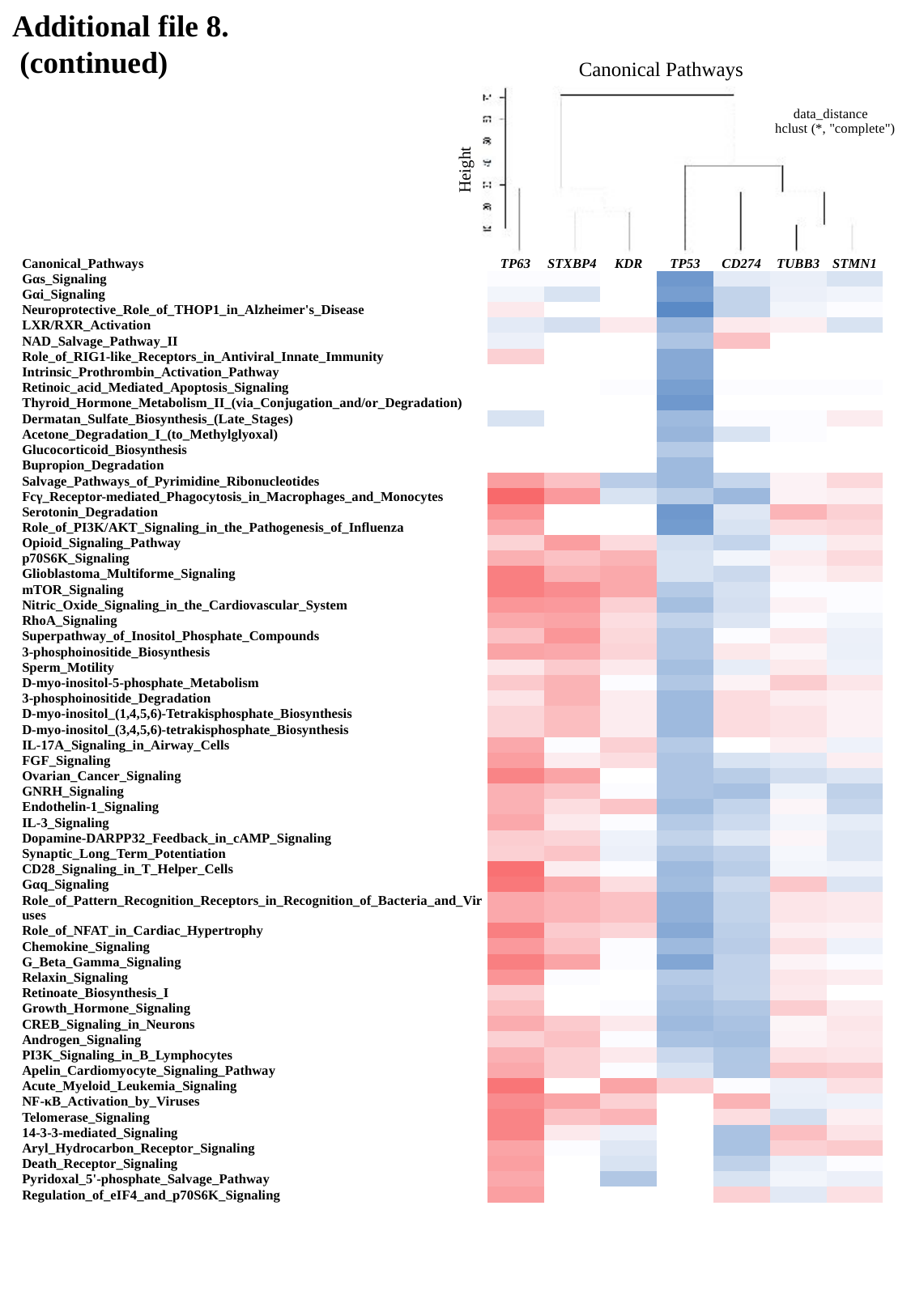

Additional file 8.
 (continued)
Canonical Pathways
data_distance
hclust (*, "complete")
Height
| Canonical\_Pathways | TP63 | STXBP4 | KDR | TP53 | CD274 | TUBB3 | STMN1 |
| --- | --- | --- | --- | --- | --- | --- | --- |
| Gαs\_Signaling | | | | | | | |
| Gαi\_Signaling | | | | | | | |
| Neuroprotective\_Role\_of\_THOP1\_in\_Alzheimer's\_Disease | | | | | | | |
| LXR/RXR\_Activation | | | | | | | |
| NAD\_Salvage\_Pathway\_II | | | | | | | |
| Role\_of\_RIG1-like\_Receptors\_in\_Antiviral\_Innate\_Immunity | | | | | | | |
| Intrinsic\_Prothrombin\_Activation\_Pathway | | | | | | | |
| Retinoic\_acid\_Mediated\_Apoptosis\_Signaling | | | | | | | |
| Thyroid\_Hormone\_Metabolism\_II\_(via\_Conjugation\_and/or\_Degradation) | | | | | | | |
| Dermatan\_Sulfate\_Biosynthesis\_(Late\_Stages) | | | | | | | |
| Acetone\_Degradation\_I\_(to\_Methylglyoxal) | | | | | | | |
| Glucocorticoid\_Biosynthesis | | | | | | | |
| Bupropion\_Degradation | | | | | | | |
| Salvage\_Pathways\_of\_Pyrimidine\_Ribonucleotides | | | | | | | |
| Fcγ\_Receptor-mediated\_Phagocytosis\_in\_Macrophages\_and\_Monocytes | | | | | | | |
| Serotonin\_Degradation | | | | | | | |
| Role\_of\_PI3K/AKT\_Signaling\_in\_the\_Pathogenesis\_of\_Influenza | | | | | | | |
| Opioid\_Signaling\_Pathway | | | | | | | |
| p70S6K\_Signaling | | | | | | | |
| Glioblastoma\_Multiforme\_Signaling | | | | | | | |
| mTOR\_Signaling | | | | | | | |
| Nitric\_Oxide\_Signaling\_in\_the\_Cardiovascular\_System | | | | | | | |
| RhoA\_Signaling | | | | | | | |
| Superpathway\_of\_Inositol\_Phosphate\_Compounds | | | | | | | |
| 3-phosphoinositide\_Biosynthesis | | | | | | | |
| Sperm\_Motility | | | | | | | |
| D-myo-inositol-5-phosphate\_Metabolism | | | | | | | |
| 3-phosphoinositide\_Degradation | | | | | | | |
| D-myo-inositol\_(1,4,5,6)-Tetrakisphosphate\_Biosynthesis | | | | | | | |
| D-myo-inositol\_(3,4,5,6)-tetrakisphosphate\_Biosynthesis | | | | | | | |
| IL-17A\_Signaling\_in\_Airway\_Cells | | | | | | | |
| FGF\_Signaling | | | | | | | |
| Ovarian\_Cancer\_Signaling | | | | | | | |
| GNRH\_Signaling | | | | | | | |
| Endothelin-1\_Signaling | | | | | | | |
| IL-3\_Signaling | | | | | | | |
| Dopamine-DARPP32\_Feedback\_in\_cAMP\_Signaling | | | | | | | |
| Synaptic\_Long\_Term\_Potentiation | | | | | | | |
| CD28\_Signaling\_in\_T\_Helper\_Cells | | | | | | | |
| Gαq\_Signaling | | | | | | | |
| Role\_of\_Pattern\_Recognition\_Receptors\_in\_Recognition\_of\_Bacteria\_and\_Viruses | | | | | | | |
| Role\_of\_NFAT\_in\_Cardiac\_Hypertrophy | | | | | | | |
| Chemokine\_Signaling | | | | | | | |
| G\_Beta\_Gamma\_Signaling | | | | | | | |
| Relaxin\_Signaling | | | | | | | |
| Retinoate\_Biosynthesis\_I | | | | | | | |
| Growth\_Hormone\_Signaling | | | | | | | |
| CREB\_Signaling\_in\_Neurons | | | | | | | |
| Androgen\_Signaling | | | | | | | |
| PI3K\_Signaling\_in\_B\_Lymphocytes | | | | | | | |
| Apelin\_Cardiomyocyte\_Signaling\_Pathway | | | | | | | |
| Acute\_Myeloid\_Leukemia\_Signaling | | | | | | | |
| NF-κB\_Activation\_by\_Viruses | | | | | | | |
| Telomerase\_Signaling | | | | | | | |
| 14-3-3-mediated\_Signaling | | | | | | | |
| Aryl\_Hydrocarbon\_Receptor\_Signaling | | | | | | | |
| Death\_Receptor\_Signaling | | | | | | | |
| Pyridoxal\_5'-phosphate\_Salvage\_Pathway | | | | | | | |
| Regulation\_of\_eIF4\_and\_p70S6K\_Signaling | | | | | | | |

## Slide 2
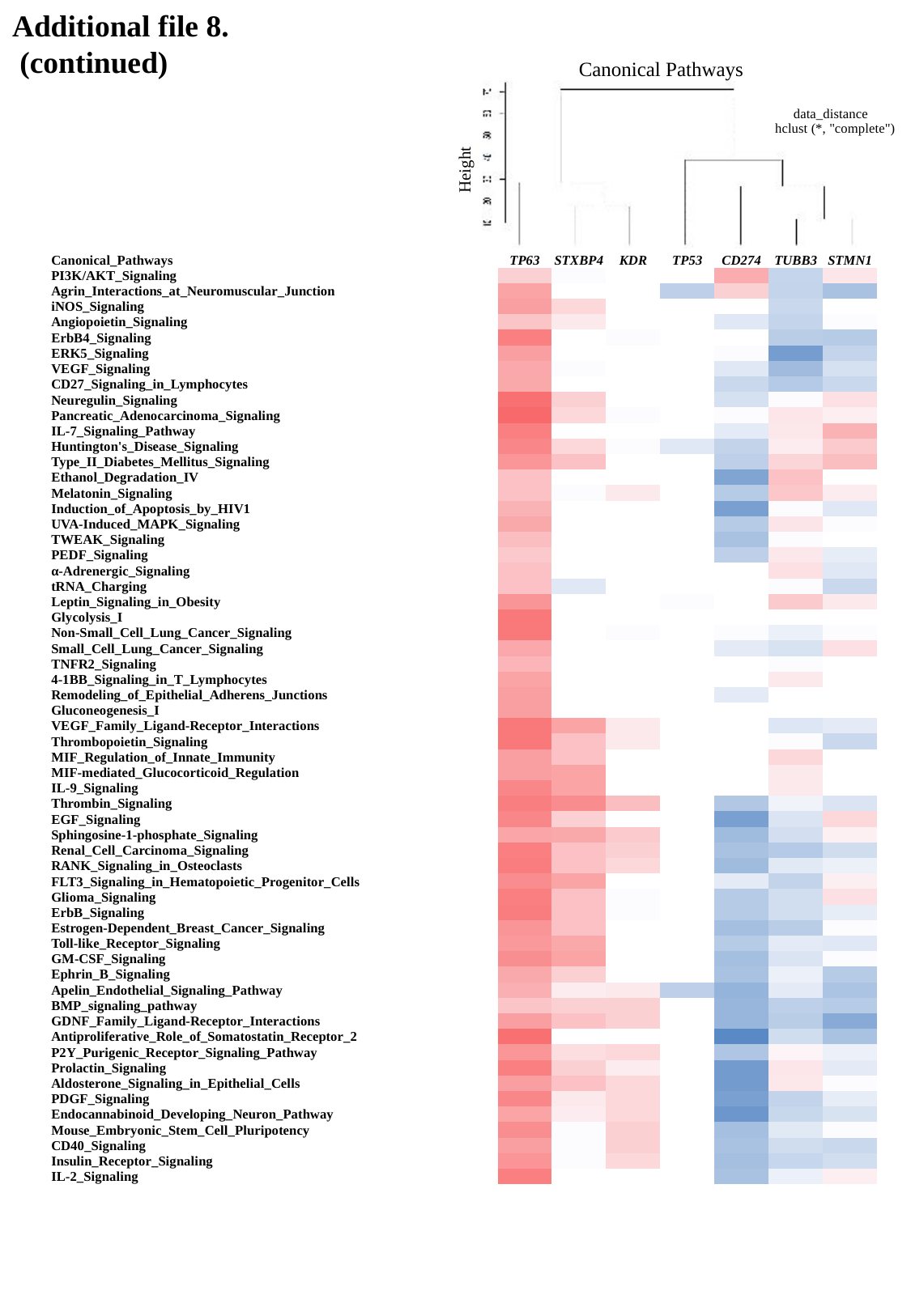

Additional file 8.
 (continued)
Canonical Pathways
data_distance
hclust (*, "complete")
Height
| Canonical\_Pathways | TP63 | STXBP4 | KDR | TP53 | CD274 | TUBB3 | STMN1 |
| --- | --- | --- | --- | --- | --- | --- | --- |
| PI3K/AKT\_Signaling | | | | | | | |
| Agrin\_Interactions\_at\_Neuromuscular\_Junction | | | | | | | |
| iNOS\_Signaling | | | | | | | |
| Angiopoietin\_Signaling | | | | | | | |
| ErbB4\_Signaling | | | | | | | |
| ERK5\_Signaling | | | | | | | |
| VEGF\_Signaling | | | | | | | |
| CD27\_Signaling\_in\_Lymphocytes | | | | | | | |
| Neuregulin\_Signaling | | | | | | | |
| Pancreatic\_Adenocarcinoma\_Signaling | | | | | | | |
| IL-7\_Signaling\_Pathway | | | | | | | |
| Huntington's\_Disease\_Signaling | | | | | | | |
| Type\_II\_Diabetes\_Mellitus\_Signaling | | | | | | | |
| Ethanol\_Degradation\_IV | | | | | | | |
| Melatonin\_Signaling | | | | | | | |
| Induction\_of\_Apoptosis\_by\_HIV1 | | | | | | | |
| UVA-Induced\_MAPK\_Signaling | | | | | | | |
| TWEAK\_Signaling | | | | | | | |
| PEDF\_Signaling | | | | | | | |
| α-Adrenergic\_Signaling | | | | | | | |
| tRNA\_Charging | | | | | | | |
| Leptin\_Signaling\_in\_Obesity | | | | | | | |
| Glycolysis\_I | | | | | | | |
| Non-Small\_Cell\_Lung\_Cancer\_Signaling | | | | | | | |
| Small\_Cell\_Lung\_Cancer\_Signaling | | | | | | | |
| TNFR2\_Signaling | | | | | | | |
| 4-1BB\_Signaling\_in\_T\_Lymphocytes | | | | | | | |
| Remodeling\_of\_Epithelial\_Adherens\_Junctions | | | | | | | |
| Gluconeogenesis\_I | | | | | | | |
| VEGF\_Family\_Ligand-Receptor\_Interactions | | | | | | | |
| Thrombopoietin\_Signaling | | | | | | | |
| MIF\_Regulation\_of\_Innate\_Immunity | | | | | | | |
| MIF-mediated\_Glucocorticoid\_Regulation | | | | | | | |
| IL-9\_Signaling | | | | | | | |
| Thrombin\_Signaling | | | | | | | |
| EGF\_Signaling | | | | | | | |
| Sphingosine-1-phosphate\_Signaling | | | | | | | |
| Renal\_Cell\_Carcinoma\_Signaling | | | | | | | |
| RANK\_Signaling\_in\_Osteoclasts | | | | | | | |
| FLT3\_Signaling\_in\_Hematopoietic\_Progenitor\_Cells | | | | | | | |
| Glioma\_Signaling | | | | | | | |
| ErbB\_Signaling | | | | | | | |
| Estrogen-Dependent\_Breast\_Cancer\_Signaling | | | | | | | |
| Toll-like\_Receptor\_Signaling | | | | | | | |
| GM-CSF\_Signaling | | | | | | | |
| Ephrin\_B\_Signaling | | | | | | | |
| Apelin\_Endothelial\_Signaling\_Pathway | | | | | | | |
| BMP\_signaling\_pathway | | | | | | | |
| GDNF\_Family\_Ligand-Receptor\_Interactions | | | | | | | |
| Antiproliferative\_Role\_of\_Somatostatin\_Receptor\_2 | | | | | | | |
| P2Y\_Purigenic\_Receptor\_Signaling\_Pathway | | | | | | | |
| Prolactin\_Signaling | | | | | | | |
| Aldosterone\_Signaling\_in\_Epithelial\_Cells | | | | | | | |
| PDGF\_Signaling | | | | | | | |
| Endocannabinoid\_Developing\_Neuron\_Pathway | | | | | | | |
| Mouse\_Embryonic\_Stem\_Cell\_Pluripotency | | | | | | | |
| CD40\_Signaling | | | | | | | |
| Insulin\_Receptor\_Signaling | | | | | | | |
| IL-2\_Signaling | | | | | | | |

## Slide 3
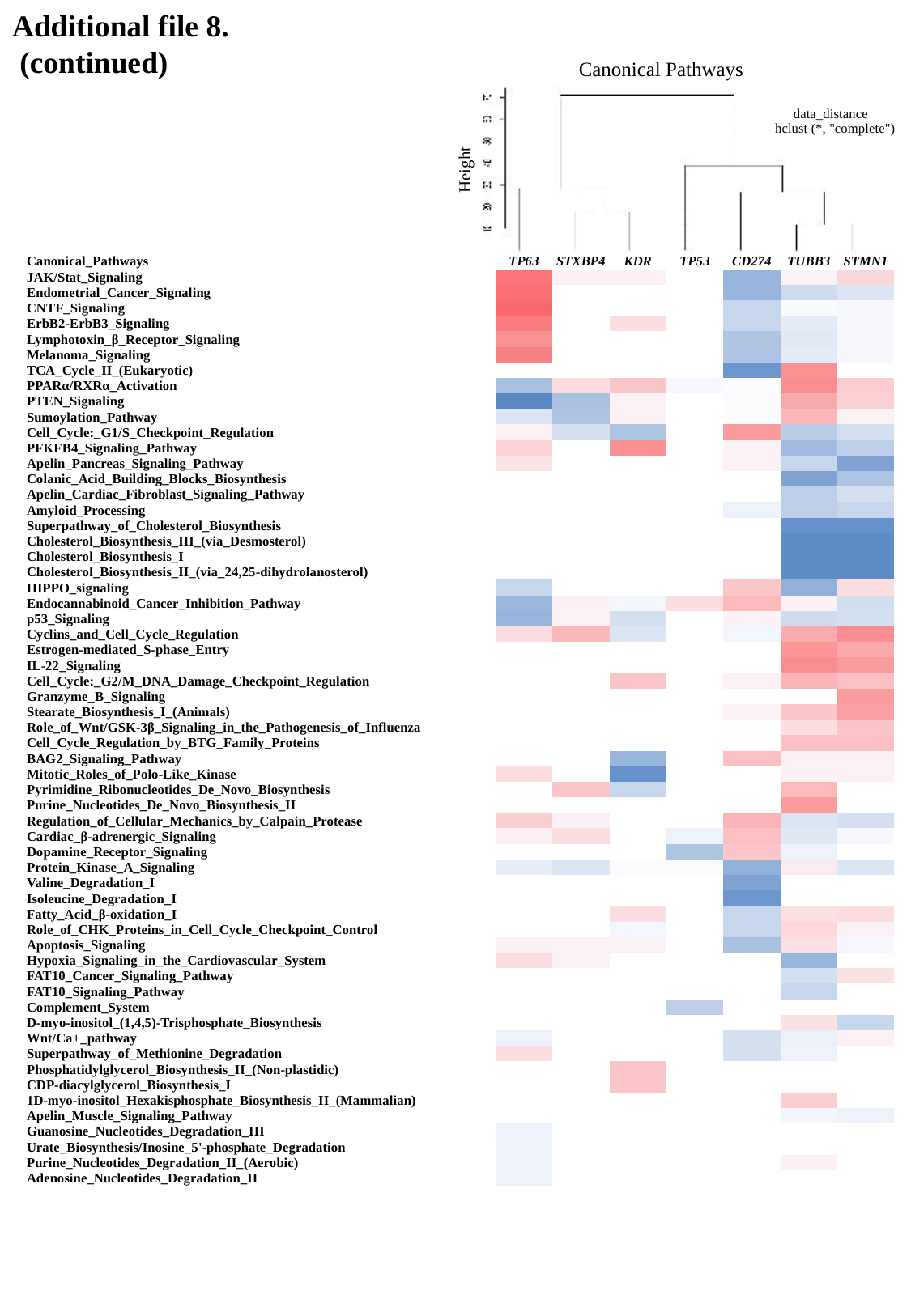

Additional file 8.
 (continued)
Canonical Pathways
data_distance
hclust (*, "complete")
Height
| Canonical\_Pathways | TP63 | STXBP4 | KDR | TP53 | CD274 | TUBB3 | STMN1 |
| --- | --- | --- | --- | --- | --- | --- | --- |
| JAK/Stat\_Signaling | | | | | | | |
| Endometrial\_Cancer\_Signaling | | | | | | | |
| CNTF\_Signaling | | | | | | | |
| ErbB2-ErbB3\_Signaling | | | | | | | |
| Lymphotoxin\_β\_Receptor\_Signaling | | | | | | | |
| Melanoma\_Signaling | | | | | | | |
| TCA\_Cycle\_II\_(Eukaryotic) | | | | | | | |
| PPARα/RXRα\_Activation | | | | | | | |
| PTEN\_Signaling | | | | | | | |
| Sumoylation\_Pathway | | | | | | | |
| Cell\_Cycle:\_G1/S\_Checkpoint\_Regulation | | | | | | | |
| PFKFB4\_Signaling\_Pathway | | | | | | | |
| Apelin\_Pancreas\_Signaling\_Pathway | | | | | | | |
| Colanic\_Acid\_Building\_Blocks\_Biosynthesis | | | | | | | |
| Apelin\_Cardiac\_Fibroblast\_Signaling\_Pathway | | | | | | | |
| Amyloid\_Processing | | | | | | | |
| Superpathway\_of\_Cholesterol\_Biosynthesis | | | | | | | |
| Cholesterol\_Biosynthesis\_III\_(via\_Desmosterol) | | | | | | | |
| Cholesterol\_Biosynthesis\_I | | | | | | | |
| Cholesterol\_Biosynthesis\_II\_(via\_24,25-dihydrolanosterol) | | | | | | | |
| HIPPO\_signaling | | | | | | | |
| Endocannabinoid\_Cancer\_Inhibition\_Pathway | | | | | | | |
| p53\_Signaling | | | | | | | |
| Cyclins\_and\_Cell\_Cycle\_Regulation | | | | | | | |
| Estrogen-mediated\_S-phase\_Entry | | | | | | | |
| IL-22\_Signaling | | | | | | | |
| Cell\_Cycle:\_G2/M\_DNA\_Damage\_Checkpoint\_Regulation | | | | | | | |
| Granzyme\_B\_Signaling | | | | | | | |
| Stearate\_Biosynthesis\_I\_(Animals) | | | | | | | |
| Role\_of\_Wnt/GSK-3β\_Signaling\_in\_the\_Pathogenesis\_of\_Influenza | | | | | | | |
| Cell\_Cycle\_Regulation\_by\_BTG\_Family\_Proteins | | | | | | | |
| BAG2\_Signaling\_Pathway | | | | | | | |
| Mitotic\_Roles\_of\_Polo-Like\_Kinase | | | | | | | |
| Pyrimidine\_Ribonucleotides\_De\_Novo\_Biosynthesis | | | | | | | |
| Purine\_Nucleotides\_De\_Novo\_Biosynthesis\_II | | | | | | | |
| Regulation\_of\_Cellular\_Mechanics\_by\_Calpain\_Protease | | | | | | | |
| Cardiac\_β-adrenergic\_Signaling | | | | | | | |
| Dopamine\_Receptor\_Signaling | | | | | | | |
| Protein\_Kinase\_A\_Signaling | | | | | | | |
| Valine\_Degradation\_I | | | | | | | |
| Isoleucine\_Degradation\_I | | | | | | | |
| Fatty\_Acid\_β-oxidation\_I | | | | | | | |
| Role\_of\_CHK\_Proteins\_in\_Cell\_Cycle\_Checkpoint\_Control | | | | | | | |
| Apoptosis\_Signaling | | | | | | | |
| Hypoxia\_Signaling\_in\_the\_Cardiovascular\_System | | | | | | | |
| FAT10\_Cancer\_Signaling\_Pathway | | | | | | | |
| FAT10\_Signaling\_Pathway | | | | | | | |
| Complement\_System | | | | | | | |
| D-myo-inositol\_(1,4,5)-Trisphosphate\_Biosynthesis | | | | | | | |
| Wnt/Ca+\_pathway | | | | | | | |
| Superpathway\_of\_Methionine\_Degradation | | | | | | | |
| Phosphatidylglycerol\_Biosynthesis\_II\_(Non-plastidic) | | | | | | | |
| CDP-diacylglycerol\_Biosynthesis\_I | | | | | | | |
| 1D-myo-inositol\_Hexakisphosphate\_Biosynthesis\_II\_(Mammalian) | | | | | | | |
| Apelin\_Muscle\_Signaling\_Pathway | | | | | | | |
| Guanosine\_Nucleotides\_Degradation\_III | | | | | | | |
| Urate\_Biosynthesis/Inosine\_5'-phosphate\_Degradation | | | | | | | |
| Purine\_Nucleotides\_Degradation\_II\_(Aerobic) | | | | | | | |
| Adenosine\_Nucleotides\_Degradation\_II | | | | | | | |
