# Supplementary material for: Distinctive roles of syntaxin binding protein 4 and its action target, TP63, in lung squamous cell carcinoma: a theranostic study for the precision medicine
Source: BMC Cancer. 2020 Sep 29;20:935. doi: 10.1186/s12885-020-07448-2 (PMC7526255; doi:10.1186/s12885-020-07448-2)
Supplement: Supplementary file 9 — Additional file 9. Thirty-five canonical pathways significantly modulated (activated or inactivated) (z-score ≥ 2) by TXT and/or Ramucirumub treatment. A totally drug-sensitive LK-2 cell line and a drug -resistant RERF-LC-AI cell line were treated with or without TXT and Ramucirumab in single and combination treatment settings, and then subjected to RNA-seq analysis. Using the gene expression data, genes highly correlated in terms of expression level with each target gene were assessed, and the 35 most significantly modulated (activated or inactivated) canonical pathways were identified. [file 12885_2020_7448_MOESM9_ESM.pptx]

## Slide 1
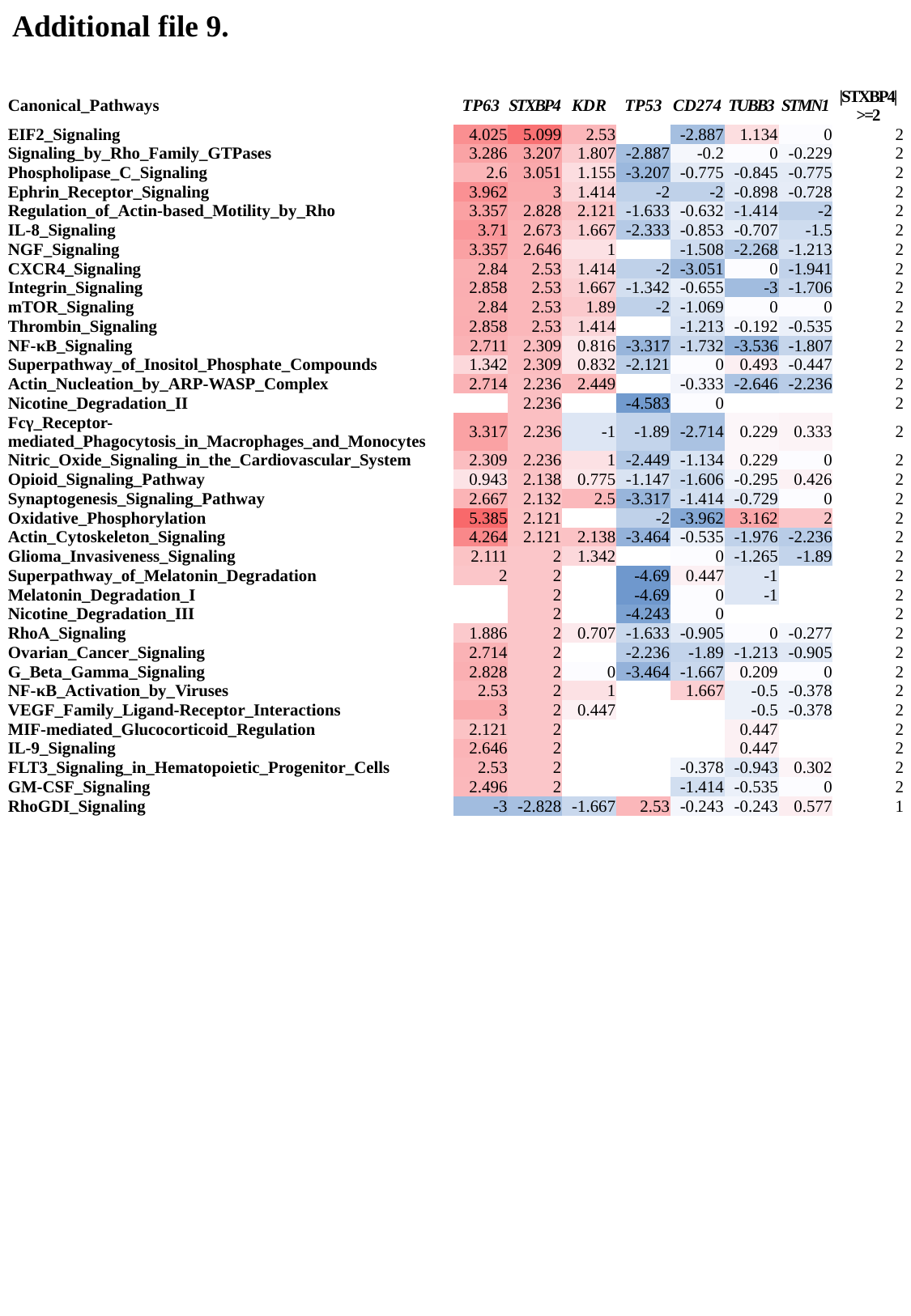

Additional file 9.
| Canonical\_Pathways | TP63 | STXBP4 | KDR | TP53 | CD274 | TUBB3 | STMN1 | |STXBP4| >=2 |
| --- | --- | --- | --- | --- | --- | --- | --- | --- |
| EIF2\_Signaling | 4.025 | 5.099 | 2.53 | | -2.887 | 1.134 | 0 | 2 |
| Signaling\_by\_Rho\_Family\_GTPases | 3.286 | 3.207 | 1.807 | -2.887 | -0.2 | 0 | -0.229 | 2 |
| Phospholipase\_C\_Signaling | 2.6 | 3.051 | 1.155 | -3.207 | -0.775 | -0.845 | -0.775 | 2 |
| Ephrin\_Receptor\_Signaling | 3.962 | 3 | 1.414 | -2 | -2 | -0.898 | -0.728 | 2 |
| Regulation\_of\_Actin-based\_Motility\_by\_Rho | 3.357 | 2.828 | 2.121 | -1.633 | -0.632 | -1.414 | -2 | 2 |
| IL-8\_Signaling | 3.71 | 2.673 | 1.667 | -2.333 | -0.853 | -0.707 | -1.5 | 2 |
| NGF\_Signaling | 3.357 | 2.646 | 1 | | -1.508 | -2.268 | -1.213 | 2 |
| CXCR4\_Signaling | 2.84 | 2.53 | 1.414 | -2 | -3.051 | 0 | -1.941 | 2 |
| Integrin\_Signaling | 2.858 | 2.53 | 1.667 | -1.342 | -0.655 | -3 | -1.706 | 2 |
| mTOR\_Signaling | 2.84 | 2.53 | 1.89 | -2 | -1.069 | 0 | 0 | 2 |
| Thrombin\_Signaling | 2.858 | 2.53 | 1.414 | | -1.213 | -0.192 | -0.535 | 2 |
| NF-κB\_Signaling | 2.711 | 2.309 | 0.816 | -3.317 | -1.732 | -3.536 | -1.807 | 2 |
| Superpathway\_of\_Inositol\_Phosphate\_Compounds | 1.342 | 2.309 | 0.832 | -2.121 | 0 | 0.493 | -0.447 | 2 |
| Actin\_Nucleation\_by\_ARP-WASP\_Complex | 2.714 | 2.236 | 2.449 | | -0.333 | -2.646 | -2.236 | 2 |
| Nicotine\_Degradation\_II | | 2.236 | | -4.583 | 0 | | | 2 |
| Fcγ\_Receptor-mediated\_Phagocytosis\_in\_Macrophages\_and\_Monocytes | 3.317 | 2.236 | -1 | -1.89 | -2.714 | 0.229 | 0.333 | 2 |
| Nitric\_Oxide\_Signaling\_in\_the\_Cardiovascular\_System | 2.309 | 2.236 | 1 | -2.449 | -1.134 | 0.229 | 0 | 2 |
| Opioid\_Signaling\_Pathway | 0.943 | 2.138 | 0.775 | -1.147 | -1.606 | -0.295 | 0.426 | 2 |
| Synaptogenesis\_Signaling\_Pathway | 2.667 | 2.132 | 2.5 | -3.317 | -1.414 | -0.729 | 0 | 2 |
| Oxidative\_Phosphorylation | 5.385 | 2.121 | | -2 | -3.962 | 3.162 | 2 | 2 |
| Actin\_Cytoskeleton\_Signaling | 4.264 | 2.121 | 2.138 | -3.464 | -0.535 | -1.976 | -2.236 | 2 |
| Glioma\_Invasiveness\_Signaling | 2.111 | 2 | 1.342 | | 0 | -1.265 | -1.89 | 2 |
| Superpathway\_of\_Melatonin\_Degradation | 2 | 2 | | -4.69 | 0.447 | -1 | | 2 |
| Melatonin\_Degradation\_I | | 2 | | -4.69 | 0 | -1 | | 2 |
| Nicotine\_Degradation\_III | | 2 | | -4.243 | 0 | | | 2 |
| RhoA\_Signaling | 1.886 | 2 | 0.707 | -1.633 | -0.905 | 0 | -0.277 | 2 |
| Ovarian\_Cancer\_Signaling | 2.714 | 2 | | -2.236 | -1.89 | -1.213 | -0.905 | 2 |
| G\_Beta\_Gamma\_Signaling | 2.828 | 2 | 0 | -3.464 | -1.667 | 0.209 | 0 | 2 |
| NF-κB\_Activation\_by\_Viruses | 2.53 | 2 | 1 | | 1.667 | -0.5 | -0.378 | 2 |
| VEGF\_Family\_Ligand-Receptor\_Interactions | 3 | 2 | 0.447 | | | -0.5 | -0.378 | 2 |
| MIF-mediated\_Glucocorticoid\_Regulation | 2.121 | 2 | | | | 0.447 | | 2 |
| IL-9\_Signaling | 2.646 | 2 | | | | 0.447 | | 2 |
| FLT3\_Signaling\_in\_Hematopoietic\_Progenitor\_Cells | 2.53 | 2 | | | -0.378 | -0.943 | 0.302 | 2 |
| GM-CSF\_Signaling | 2.496 | 2 | | | -1.414 | -0.535 | 0 | 2 |
| RhoGDI\_Signaling | -3 | -2.828 | -1.667 | 2.53 | -0.243 | -0.243 | 0.577 | 1 |
